# Heterologous investigation of metabotropic and ionotropic odorant receptors in ab3A neurons of *Drosophila melanogaster*

**DOI:** 10.3389/fmolb.2023.1275901

**Published:** 2024-01-25

**Authors:** Johan Henning Pettersson, Alberto Maria Cattaneo

**Affiliations:** ^1^ Department of Plant Protection Biology, Chemical Ecology Group, Swedish University of Agricultural Sciences, Lomma, Sweden; ^2^ Benton Lab, Center for Integrative Genomics, University of Lausanne, Lausanne, Switzerland; ^3^ Whitney Laboratory for Marine Bioscience, University of Florida, Gainesville, FL, United States

**Keywords:** metabotropic receptors (mGluRs), ionotropic receptors, heterologous expression, transgenic *Drosophila melanogaster*, empty ab3A neurons, single sensillum recording, functional characterization of chemoreceptors, deorphanization

## Abstract

In insects, antennal ionotropic receptors (IRs) and odorant receptors (ORs) are among the main sensors of olfactory cues. To functionally characterize the subunits from these receptors, the use of ab3A neurons from transgenic *Drosophila melanogaster* represented one of the most powerful tools, allowing the identification of ligands (deorphanization) and decrypting their pharmacological properties. However, further investigation is needed to shed light on possible metabotropic functionalities behind insect olfactory receptors and test potentials from the up-to-now-used *empty neuronal systems* to express subunits belonging to variegate receptor classes. In this project, we adopted the most updated system of *Drosophila* ab3A empty neurons to test various olfactory receptors, ranging from human ORs working as metabotropic G-protein coupled receptors to insect ionotropic IRs and ORs. Testing transgenic *Drosophila* expressing human ORs into ab3A neurons by single sensillum recording did not result in an OR response to ligands, but it rather re-established neuronal spiking from the empty neurons. When transgenic *D. melanogaster* expressed ionotropic IRs and ORs, both heterologous and cis-expressed IRs were non-functional, but the *Drosophila suzukii* OR19A1 subunit responded to a wide asset of ligands, distinguishing phasic or tonic compound-dependent effects. Despite the use of *Drosophila* ab3A neurons to test the activation of some metabotropic and ionotropic receptor subunits resulted non-functional, this study deorphanized a key OR of *D. suzukii* demonstrating its binding to alcohols, ketones, terpenes, and esters.

## Introduction

Among the chemosensory receptors of insects, odorant receptors (ORs) and ionotropic receptors (IRs) represent the majority of subunits expressed in their antennal neurons, whose major role is the transduction of chemical signals from the environment (Wicher and Miazzi, 2021).

Since the discovery of ORs in mammals (Buck and Axel, 1991), which demonstrated their working as G-protein-coupled receptors (GPCRs), evidence from the inverted topology of the subunits of insects (Benton et al., 2006) instead suggested their ionotropic functionality. The absence of an intracellular C-terminal domain excluded the triggering of G-protein-mediated metabotropic mechanisms (Conklin et al., 1996). Furthermore, in insect olfactory sensory neurons (OSNs), ORs are co-expressed together with a highly conserved olfactory co-receptor (Larsson et al., 2004; Pitts et al., 2004; Corcoran et al., 2014), forming functional heterotetramers working as ligand-gated ion channels (Butterwick et al., 2018). More direct pieces of evidence for ligand-gated channel properties of insect ORs were obtained performing outside-out patch-clamp recording when Orco + OR complexes were co-expressed either in oocytes from *Xenopus* or in human embryonic kidney (HEK293T) cells (Sato et al., 2008; Cattaneo et al., 2017b).

In a more complex scenario, several G-protein alpha subunits, in particular, Gαq, have been found expressed in insect antennae (Talluri et al., 1995; Laue et al., 1997; Miura et al., 2005; Yao and Carlson, 2010; Raja et al., 2014). Functional studies reported the deorphanization of insect ORs when heterologously co-expressed with α-subunits of G-proteins in mammalian cell systems (Grosse-Wilde et al., 2007; Grosse-Wilde et al., 2006). In addition, evidence from electrophysiological investigations on *Drosophila melanogaster* proposed insect Orco + OR channels working as metabotropically regulated ionotropic receptors (Getahun et al., 2013), modulated by enzymes, or cyclic-AMP-related mechanisms, downstream of G-protein activation of the insect’s olfactory systems (Riesgo-Escovar et al., 1995; Gomez-Diaz et al., 2004; Gomez-Diaz et al., 2006a; Gomez-Diaz et al., 2006b; Kain et al., 2008; Deng et al., 2011; Chatterjee et al., 2009).

Contrary to insect ORs, expression of which is limited to insects (Wicher and Miazzi, 2021), the ionotropic receptors (IRs) represent a class of transmembrane chemoreceptors expressed in the sensory neurons of different animals belonging to the superphylum Protostomia, working as ligand-gated ion channels (Croset et al., 2010; Eyun et al., 2017). IRs are evolutionarily related to the ionotropic glutamate receptors (iGluRs), an ancient class of chemosensors involved in synaptic and post-synaptic neuronal communication, expressed among various organisms from the whole animal kingdom (Gereau and Swanson, 2008) to a small number of prokaryotes (Chen et al., 1999; Chiu et al., 1999) and plants (Lam et al., 1998). Since their discovery (Benton et al., 2009), IRs have been investigated mostly in insects (Silbering et al., 2011; Hussain et al., 2016; Herre et al., 2022), where they diverged for their involvement in olfaction (antennal IRs (A-IRs), Croset et al., 2010), taste (Stewart et al., 2015; divergent IRs (D-IRs), Koh et al. (2014); Tauber et al. (2017)), or other sensory modalities, including the detection of CO_2_ (Ai et al., 2013) and heat and humidity (van Giesen and Garrity, 2017).

For both ORs and IRs, recording *in vivo* on neurons from transgenic *D. melanogaster* represented one of the main milestones of success in the functional characterization of insect chemoreceptors (Dobritsa et al., 2003). In specific, performing single sensillum recording (SSR) on ab3A heterologously expressing different OR subunits decrypted some key chemosensory mechanisms of various insects, including pests, for human health (Wheelwright et al., 2021), human activities (Montagne et al., 2012; Bengtsson et al., 2014; Cattaneo et al., 2017b; de Fouchier et al., 2017; Cattaneo et al., 2022), or simple human commensals (Lebreton et al., 2017). Despite studies on IRs in ab3As being limited, misexpression of ab3A neurons of IR subunits from *D. melanogaster* demonstrated that co-expression with specific co-receptors is sufficient to form functional ion channels capable of conferring ab3A responsiveness to specific odors (Abuin et al., 2011).

In this study, we attempted to shed further light on possible metabotropic aspects behind the chemosensory transduction of insect olfactory neurons. Using the most recently engineered *D. melanogaster* lines expressing Gal4 into empty ab3A neurons (Chahda et al., 2019), we performed SSR to compare the functional activation of three classes of chemosensors from different organisms: mammalian ORs (GPCRs), among which we selected subunits from humans, and insect IRs and ORs, working as the ligand-gated cation channels. Although we recognize that attempting the expression and functional characterization of GPCRs using insect OSNs may be challenging, we justified the choice of this approach based on previous evidence of G-protein expression within *D. melanogaster* neurons (Talluri et al., 1995; Yao and Carlson, 2010; Raja et al., 2014).

Despite the heterologous expression of mammalian GPCR-associated ab3A spiking, lack of activation from these and the IR subunits suggested that the use of ab3A neurons would be more suitable to design the expression of insect ORs working as ligand-gated cation channels. To test this hypothesis, we chose two ORs from the spotted wing *Drosophila suzukii*, a key pest of horticulture worldwide, on which our labs have recently invested significant efforts to decrypt its molecular bases of olfaction (Cattaneo et al., 2022; Walker et al., 2023). In particular, we selected DsuzOR19A1 and DsuzOR19A2 orthologs from the *D. melanogaster* OR19a, which is renowned as a terpenes sensor reflecting both oviposition preference and parasitoids avoidance (Dweck et al., 2013). Using *D. melanogaster* ab3A neurons, out of these two ORs, we were able to deorphanize DsuzOR19A1. The lack of effects when we attempted the deorphanization of DsuzOR19A2 underlined further limitations of the ab3A empty neuron system that were already encountered in previous studies testing different ORs channels (Montagne et al., 2012; Bengtsson et al., 2014; Ronderos et al., 2014).

While attempting the expression of metabotropic and ionotropic receptors in *D. melanogaster* ab3A neurons resulted non-functional, this work adds to the functional characterization results of *D. suzukii* ORs and highlights the importance of an accurate choice of *Drosophila* neuronal systems in the design of functional studies for specific chemoreceptor subunits.

## Materials and methods

### Insects

Transgenic *D. melanogaster* were maintained in our facilities on a sugar–yeast–cornmeal diet (https://bdsc.indiana.edu/information/recipes/bloomfood.html) at room temperature (25°C ± 2°C) and a relative humidity of 50% ± 5% under a 12:12 light:dark photoperiod.

### Amplification and cloning

The coding sequences of human ORs were amplified, starting from pCI-OR1a1 (AddGene #22319) and pCI-OR2W1 (AddGene # 21686), which were kindly provided by Dr. Yuriy V. Bobkov (Whitney Laboratory for Marine Bioscience, University of Florida). Primers were designed to amplify the complete ORFs based on the respective deposited sequences (HsapOR1a1, GenBank NM_001386104.1: forward: 5′- ATG​GAC​CAA​AGC​AAT​TAT​AGT​TC-3’, reverse: 5′-CTA​TGA​CTT​GCA​ATT​CCT​C-3’; HsapOR2W1, GenBank KY500511.1: forward: 5′-ATG​GAT​ATT​GTG​GAG​GTG​GAC-3’, reverse: 5′-TTA​GTG​GCT​TTC​ATT​AGT​AG-3′). The coding sequences of *D. suzukii* OR19As were amplified starting from *D. suzukii* retro-transcribed cDNA templates derived from total RNA samples obtained as described by Cattaneo et al. (2022). Primers were designed to amplify complete ORFs based on the deposited data from the work of Ramasamy et al. (2016) (DsuzOR19A1, forward: 5′-CAC​CAT​GGA​TAT​TGT​GGA​GGT​GGA​C-3’, reverse: 5′-TTA​GTG​GCT​TTC​ATT​AGT​AG-3’; DsuzOR19A2, forward: 5′-CAC​CAT​GGA​AAT​TCA​GAA​GGT​GGA​T-3’, reverse: 5′-TTA​GTG​ACT​TTC​AAG​AGT​CG-3′). Primers’ melting temperatures were calculated using the salt-adjusted algorithm of OligoCalc (http://biotools.nubic.northwestern.edu/OligoCalc.html) using the average melting temperatures as annealing temperatures (Tm) for primer pairs in the phase of amplification. Amplifications were performed with Advantage 2 polymerase (Clontech, Mountain View, CA, United States of America), setting a temperature program of 94°C for 1 min, followed by 35 cycles of 94°C for 30 s, Tm for 30 s, and 68°C for 1 min. A final extension step at 68°C for 7 min concluded the reaction. DNA amplicons were purified on agarose gel. Cloning into PCR8/TOPO (Invitrogen Life Technologies, Grand Island, NY, United States) was performed following the manufacturer’s protocol upon a short adenylating cycle using dATP and polymerase from the Advantage kit. To test the correct orientation in the PCR8/TOPO vector, colonies were screened by performing PCR, combining the universal M13 forward primer with gene-specific reverse primers. Plasmids were extracted according to the protocols using the ZR Plasmid Miniprep kit (Zymo Research, Irvine, CA, United States). The integrity and orientation of the insert were confirmed by Sanger sequencing 3730xl (Eurofins Genomics, Ebersberg, Germany). Cassettes with inserts were then transferred from their PCR8/GW/TOPO plasmids to the destination vector (pUASg-HA.attB, constructed by E. Furger and J. Bischof, kindly provided by the Basler group, Zürich, Switzerland) using the Gateway LR Clonase II kit (Invitrogen). Sanger sequencing checked the integrity and orientation of the inserts further. Expression vectors containing pUAS-CpomIR64a and pUAS-DsuzIR64a cassettes were generated in the frame of another project (Cattaneo et al., 2023).

To perform single-fly PCR (Supplementary Figure S1), Advantage 2 polymerase was combined with primers designed on the UAS promoter or on Gal4/DSRed genes (Supplementary Table S1). Kinetic settings were adjusted based on the primers’ melting temperatures and amplicon lengths, considering 1 min as the amplification time for 1,000 bp. Amplifications were conducted from single-fly genomic templates obtained by smashing insects directly into the squishing buffer (10 mM Tris-HCl; 1.0 mM EDTA; and 25 mM NaCl [pH 7.5]) combined with 200 μg/mL Proteinase K (Thermo Fisher Scientific, Waltham, MA, USA), incubating smashed insects at 37°C for 30 min, and heating at 95°C for 5 min.

### Fluorescent *in situ* hybridization

FISH was performed, as recently reported by Cattaneo et al. (2022). In specific, for this hybridization, we used single own-synthesized DIG starting from linearized pCR8 vectors containing DsuzOR19A1/2-coding sequences. A total of 1.5 μg of DNA vector was linearized with BbsI following the recommended protocols (New England Biolabs, Ipswich, MA, United States) to be purified in RNAse-free water and checked on agarose gel electrophoresis to verify the linearization of the plasmids. One-third of the purified volume (∼0.5 μg) was amplified with T7-RNA polymerase (Promega, Madison, WI, United States) integrating DIG-labeled ribonucleotides (BMB Cat. #1277073, Roche, Basel, Switzerland) following the recommended protocols (https://www.rockefeller.edu/research/uploads/www.rockefeller.edu/sites/8/2018/10/FISHProtocolKSVRevised.pdf). *D. suzukii* antennae were collected from male and female adult insects from our rearing facility (FORMAS Swedish Research Council, project numbers 2011-390 and 2015-1221). RNA FISH on whole-mount antenna was conducted as described by Saina and Benton (2013) by staining with a single probe for each experiment. Imaging was performed on a Zeiss confocal microscope LSM710 using a ×40 immersion objective; settings were adjusted based on single antenna: DIG-labeled probes staining specific neurons were visualized setting Cy5-laser between 4% and 10% and calibrating gain in a range of 700–900. Staining was conducted in parallel with control experiments already reported in the work of Cattaneo et al. (2022) using IR60b and Orco as the negative and positive controls. Neuronal counting was performed using the cell-counter tool of ImageJ. To identify the differences between males and females, DsuzOR19A1 neuron numbers were compared with a heteroscedastic one-tailed two-sample *t*-test (ɑ = 0.05) upon conducting tests of normality with R and the IBM SPSS Statistics software 29.0 (https://www.ibm.com/). We decided to choose a heteroscedastic two-sample *t*-test because Levene’s test of homogeneity (ɑ = 0.05) demonstrated that variances between male and female groups were not equal [IBM SPSS Statistics 29.0, nominal variable: genders (group 1: male; group 2: female) and scale variable: neuronal count]. Tests of normality for the neuronal count of DsuzOR19A2 unveiled significant differences from normal distributions: neuron numbers between males and females were compared with a non-parametric independent Mann–Whitney *U*-test.

### Heterologous expression in *Drosophila melanogaster* ab3A neurons

Transformant lines for *pUAS-HsapOR1a1*, *pUAS-HsapOR2W1*, *pUAS-DsuzOR19A1*, *pUAS-DsuzOR19A2*, *pUAS-CpomIR64a*, and *pUAS-DsuzIR64a* were generated by Best Gene (Chino Hills, CA, United States) through PhiC31 standard integration. For *pUAS-HsapOR1a1*, integration targeted the X chromosome by injecting BDSC#32233 and BDSC#32107 strains; for all the other constructs, integration targeted the third chromosome, injecting BDSC#8622 strains. Crossings were performed with balancer lines in accordance with procedures already published from our labs (Gonzalez et al., 2016).

To express human and insect OR transgenes into ab3A neurons, parental strains with the Gal4 gene knocked into the OR22a/b-locus (*w;pOR22a-Gal4* ^*KI*^ *:+* - Chahda et al. (2019)) were kindly provided by Prof. John Carlson (Dept. of Molecular Cellular and Developmental Biology, Yale University). This strain was used for crossing, as shown in Supplementary Figure S1A, to generate the following genotypes: *w,pUAS-* ***HsapOR1a1*** *;pOR22a-Gal4* ^*KI*^ *;+*; *w,pOR22a-Gal4* ^*KI*^ *;* ***pUAS-HsapOR2W1***; *w,pOR22a-Gal4* ^*KI*^ *;* ***pUAS-DsuzOR19A1***; and *w,pOR22a-Gal4* ^*KI*^ *;* ***pUAS-DsuzOR19A2***. From these results, an additional crossing was performed to obtain a genotype combining both human ORs: *w,* ***pUAS-HsapOR1a1*** *;pOR22a-Gal4* ^*KI*^ *:* ***pUAS-HsapOR2W1***.

To express *CpomIR64a* and *DsuzIR64a* into ab3A neurons, parental *pOR22a-Gal4* ^*KI*^ lines were first recombined with *pUAS-IR8a* lines (BDSC#41745) to co-express the *D. melanogaster* IR8a co-receptor (Benton et al., 2009). IR8a is well known for forming cation channels with IR64as (Ai et al., 2010; Ai et al., 2013) and other acid sensors, such as IR84a (Abuin et al., 2011). Following procedures described above for single-fly PCR screening, we selected recombinants on the second chromosome (Supplementary Figure S1B). Choosing to *pUAS*-co-express IR8a was motivated by the importance of this co-receptor subunit in forming cation channels with IR64a-sensors (Benton et al., 2009; Ai et al., 2013) and by the functional evidence from this approach from previous research on a different IR of *D. melanogaster* (Abuin et al., 2011). Homozygous lines from these recombinant flies (*w,pUAS-IR8a,pOR22a-Gal4* ^*KI*^) were crossed with balanced *pUAS-Cpom/DsuzIR64a* flies to generate *w,pUAS-IR8a,pOR22a-Gal4* ^*KI*^ *;* ***pUAS-CpomIR64a*** and *w,pUAS-IR8a,pOR22a-Gal4* ^*KI*^ *;* ***pUAS-DsuzIR64a***. Further crossings were conducted combining *pUAS-Cpom/DsuzIR64a*-flies with a further *pUAS-IR8a* line with insertion in the X chromosome (*w,pUAS-IR8a;Bl/CyO;TM2/TM6B*—a gift from Prof. Richard Benton, Center for Integrative Genomics, University of Lausanne) and with *pOR22a-Gal4* ^*KI*^ to obtain the following genotypes: *w,pUAS-IR8a;pOR22a-Gal4* ^*KI*^ *;* ***pUAS-CpomIR64a*** and *w,pUAS-IR8a;pOR22a-Gal4* ^*KI*^ *;* ***pUAS-DsuzIR64a***.

Further crossings were performed with the latter using *pUAS-IR84a* lines (BDSC#41740) to obtain *w,pUAS-IR8a,pOR22a-Gal4* ^*KI*^ */* ***pUAS-IR84a*** and *w,pUAS-IR8a;pOR22a-Gal4* ^*KI*^ */* ***pUAS-IR84a***.

To test Gal4 functionality through immunostaining, parental *pOR22a-Gal4* ^*KI*^ lines were crossed with *w,pUAS-nGFP;+* (kindly provided by Prof. Richard Benton) selecting *pOR22a-Gal4* ^*KI*^/*pUAS-nGFP* heterozygous.

To express DsuzOR19A2 in *∆halo Drosophila* for supplementary GC-SSR experiments, we crossed *w;Δhalo/CyO;pOr22a-Gal4* mutant lines (Dobritsa et al., 2003) with a parallel transformant obtained upon crossing *pUAS-DsuzOR19A2* with *∆halo* (*w;Δhalo/CyO;pUAS-DsuzOR19A2*) to a final genotype *w;Δhalo/CyO;pOr22a-Gal4/pUAS-DsuzOR19A2*.

### Immunostaining

Immunostaining was performed on *D. melanogaster* antennae collected from female adult insects from our rearing facility (genotype: w;*pOR22a-Gal4* ^*KI*^/*pUAS-nGFP;+*, see the previous section) following similar protocols described by Saina and Benton (2013). Protocols were adjusted using rabbit anti-GFP 1:1,000 as a primary antibody and Alexa anti-rabbit 1:100 as a secondary antibody. Imaging was performed on a Zeiss confocal microscope LSM710 using a ×40 immersion objective. The settings were adjusted based on single antenna, and neurons were visualized using 488-laser at 4%, gain 700–900. Images were analyzed, split, and elaborated using ImageJ (Fiji, https://imagej.net/ij/).

### Single sensillum recording

Heterologous subunits or their combinations expressed in neurons of ab3 sensilla were tested through single sensillum recordings (SSR), as we performed in recent studies (Cattaneo et al., 2022). In brief, 3–8-day-old flies were immobilized in 100 μL pipette tips with only the top half of the head protruding. The right antenna of each insect was gently pushed with a glass capillary against a piece of glass. This piece of glass and the pipette tip were fixed with dental wax on a microscope slide. Electrolytically sharpened tungsten electrodes (Harvard Apparatus Ltd., Edenbridge, United Kingdom) were used to penetrate the insect’s body: the reference electrode was manually inserted into the right eye of the fly, while the recording electrode was maneuvered with a DC-3K micromanipulator equipped with a PM-10 piezo translator (Märzhäuser Wetzler GmbH, Wetzler, Germany) and inserted in the ab3 sensilla. signals coming from the olfactory sensory neurons were amplified 10 times with a probe (INR-02, Syntech, Hilversum, the Netherlands), digitally converted through an IDAC-4-USB (Syntech) interface, and visualized and analyzed with the software AutoSpike v. 3.4 (Syntech). To carry the odorant stimulus, prevent antennal dryness, and minimize the influence of background odors from the environment, a constant humidified flow of 2.5 L/min charcoal-filtered air was delivered through a glass tube and directed toward the preparation.

Stimuli (Table 1) were diluted in hexane, ethanol (Sigma-Aldrich, St. Louis, MO, USA), or water to prepare stimulus with 2.5 μL of 10 μg/μL dilutions. As carried out by Cattaneo et al. (2022), the choice of ab3 sensilla was based on testing the ab3B-specific ligands 3-octanol (CAS: 589-98-0) and 2-heptanone (CAS 110-43-0) and the ab2-specific ethyl acetate (CAS: 141-78-6). Ethyl hexanoate (CAS 123-66-0) was also included in the panel to test a possible residual expression of OR22a/b in transgenic lines. Stimuli aliquots were spread on grade 1–20 mm circle filter paper (GE Healthcare Life Science, Little Chalfont, United Kingdom), previously inserted into glass Pasteur pipettes (VWR, Milan, Italy). To minimize possible effects from the solvent, pipettes were let at least 10 min after preparation under the fume hood for solvent evaporation. Puffing provided additional 2.5 mL of air through the pipette for 0.5 s by inserting the pipette within a side hole of the glass tube directing the humidified airflow to the antennae. Responses to compounds of the panel were compared for three to six replicates depending on the experiment, using a single insect as a replicate. To characterize the intensity of the response, spike frequency was calculated as carried out by Lebreton et al. (2017) and Cattaneo et al. (2022) by subtracting the ab3A spikes counted for 1.0 s before the stimulus from the number of spikes counted for 1.0 s after the stimulus, with the aim to calculate the spike frequency in terms of Δspikes/sec. Finally, to validate significant differences in spike counting, a non-parametric paired Wilcoxon signed-rank test, using IBM SPSS Statistics software 29.0, compared the spike frequencies enhanced by the respective solvents with the spike frequency associated to each compound.

**TABLE 1:** Panel of ligands tested on transgenic *D. melanogaster*.

Compound	Compound class	CAS	Molecular weight (g/mol)	Vapor pressure (mmHg @ 20°C–25°C)	Solvent	Receptor target	Additional experiments	
							GC-SSR	HEK293T
1-Octanol	Primary alcohol	111-87-5	130,2307	0,07940	Ethanol	IR64as		
3-Octanol	Secondary alcohol	589-98-0	130,2300	0,51200	Hexane	OR85b (+)		
Nerol	Monoterpenoid alcohol	106-25-2	154,2500	0,01300	Ethanol	DsuzORs		
Farnesol	Acyclic sesquiterpene alcohol	4602-84-0	222,3714	0,00037	Ethanol	DsuzORs		
2-Heptanone	Aliphatic chetone	110-43-0	114,1878	4,73200	Hexane	OR85b (+)		
R-carvone	Monoterpenoid chetone	99-49-0	150,2208	0,16000	Hexane	HsapGPCRs		
S-carvone	Monoterpenoid chetone	99-49-0	150,2208	0,16000	Hexane	HsapGPCRs		
Nonanal	Insaturated aldehyde	124-19-6	142,2417	0,53200	Hexane	HsapGPCRs		
(E,E)-2,4-decadienal	Polyinsaturated aldehyde	25,152-84-5	152,2367	0,03000	Hexane	HsapGPCRs		
(Z)-3-nonenal	Monoinsaturated aldehyde	31,823-43-5	140,2257	0,39600	Hexane	HsapGPCRs		
(Z)-4-nonenal	Monoinsaturated aldehyde	2277-15-8	140,2257	0,39800	Hexane	HsapGPCRs		
(Z)-4-undecenal	Monoinsaturated aldehyde	68820-32-6	168,2796	0,04500	Hexane	HsapGPCRs		
(Z)-6-undecenal	Monoinsaturated aldehyde	60671-73-0	168,2796	0,04540	Hexane	HsapGPCRs		
Cuminaldeide	Aromatic aldehyde	122-03-2	148,2020	0,04820	Ethanol	DsuzORs		
Phenylacetaldehyde	Aromatic aldehyde	122-78-1	120,1500	0,39000	Water	IR64as		
Benzaldehyde	Aromatic aldehyde	100-52-7	106,1240	1,27000	Ethanol	IR64as		
Citral	Acyclic monoterpene aldehyde	5392-40-5	152,2400	0,20000	Ethanol (#DMSO)	DsuzORs		
Ethyl hexanoate	Fatty acid ester	123-66-0	144,2139	1,66500	Hexane	OR22a (+)		
Ethyl acetate	Acetate ester	141-78-6	88,1100	111,71600	Hexane	DsuzORs		
Bornyl acetate	Acetate ester	76-49-3	196,2898	0,22800	Ethanol	DsuzORs		
m-Cymene	Monoterpene	535-77-3	134,2200	1,72000	Ethanol	DsuzORs		
p-Cymene	Monoterpene	99-87-6	134,2216	1,46000	Ethanol	DsuzORs		
α-Pinene	Monoterpene	80-56-8	136,2300	4,75000	Ethanol	DsuzORs		
β-Pinene	Monoterpene	127-91-3	136,2300	2,93000	Ethanol	DsuzORs		
Limonene oxide	Monoterpene	1195-92-2	152,2367	0,51500	Ethanol	DsuzORs		
α-Terpinene	Monoterpene	99-86-5	136,2380	1,63800	Ethanol	DsuzORs		
Ocimene	Monoterpene	3779-61-1	136,2340	1,55900	Ethanol	DsuzORs		
γ-Terpinene	Monoterpene	99-85-4	136,2375	1,07500	Ethanol	DsuzORs		
β-Myrcene	Monoterpene	123-35-3	136,2380	2,29000	Ethanol	DsuzORs		
Camphene	Monoterpene	5794-03-6	136,2380	3,38000	Ethanol	DsuzORs		
Terpinolene	Monoterpene	586-62-9	136,2300	1,12600	Ethanol	DsuzORs		
beta Citronellol	Monoterpenoid	106-22-9	157,2700	0,02000	Ethanol	IR64as		
α-Phellandrene	Cyclic monoterpene	99-83-2	136,2400	1,85600	Ethanol	DsuzORs		
R-limonene	Cyclic monoterpene	5989-27-5	136,2380	0,19800	Ethanol	DsuzORs		
S-limonene	Cyclic monoterpene	5989-54-8	136,2380	1,54100	Ethanol	DsuzORs		
3-Carene	Bicyclic monoterpene	13466-78-9	136,2380	3,72000	Ethanol	DsuzORs		
α-Humulene	Monocyclic sesquiterpene	6753-98-6	204,3563	0,08000	Ethanol	DsuzORs		
β-Caryophiillene	Bicyclic sesquiterpene	87-44-5	204,3570	0,01300	Ethanol	DsuzORs		
α-Cedrene	Bicyclic sesquiterpene	469-61-4	204,3570	0,01800	Ethanol	DsuzORs		
β-Cedrene	Bicyclic sesquiterpene	546-28-1	204,3570	0,01700	Ethanol	DsuzORs		
Valencene	Bicyclic sesquiterpene	4630-07-03	204,3570	0,01100	Ethanol	DsuzORs		
β-Caryophiillene oxide	Sesquiterpenoid	1139-30-6	220,3555	0,00700	Ethanol	DsuzORs		
Ammonium hydroxide	Non-metal hydroxide	1336-21-6	35,0460	2160,00000	Water	IR64as		
Ammonia	Pnictogen hydride	7664-41-7	17,0310	7500,00000	Water	IR64as		
Hexylamine	Primary amine	111-26-2	101,1930	7,95000	Water	IR64as		
Buthylamine	Primary amine	109-73-9	73,1390	92,90000	Water	IR64as		
Pyrrolidine	Secondary amine	123-75-17	71,1200	62,70000	Water	IR64as		
Dimethylamine	Secondary amine	124-40-3	45,0850	1520,00000	Water	IR64as		
Triethylamine	Tertiary amine	121-44-8	101,1930	57,07000	Water	IR64as		
Pyridine	Tertiary amine	110-86-1	79,1000	20,80000	Ethanol	IR64as		
Putrescine	Diamine	110-60-1	88,1500	2,33000	Water	IR64as		
Cadaverine	Diamine	462-94-2	102,1810	1,01000	Water	IR64as		
Spermidine	Polyamine	124-20-9	145,2500	0,00100	Water	IR64as		
2-Phenyletylamine	Aromatic amine	64-04-0	121,1830	0,23000	Ethanol	IR64as		
Formic acid	Carboxylic acid	64-18-6	46,0250	36,47700	Water	IR64as		
Acetic acid	Carboxylic acid	64-19-76	60,0520	15,70000	Water	IR64as		
Propionic acid	Carboxylic acid	79-09-4	74,0790	3,53000	Water	IR64as		
Butanoic acid	Carboxylic acid	107-92-6	88,1060	1,65000	Water	IR64as		
Hexanoic acid	Carboxylic acid	142-62-1	116,1600	0,04350	Water	IR64as		
Octanoi acid	Carboxylic acid	124-07-2	144,2140	0,00371	Water	IR64as		
Phenylaceic acid	Aromatic carboxylic acid	103-82-2	136,1500	0,00380	Water	IR84a		
VUAA1	Acetamide	52,5582-84-7	367,4700	No data available	DMSO	Orco (+DsuzORs)		
Water	Oxygen hydride	7732-18-5	18,0150	24,47500	-	Solvent		
Ethanol	Primary alcohol	64-17-5	40,0690	59,30000	-	Solvent		
Hexane	Alkane	110-54-3	86,1776	151,00000	-	Solvent		
DMSO	Organosulfur	67-68-5	78,1340	0,61000	-	Solvent		

Synthetic ligands were tested on the different receptor subunits expressed in ab3A neurons. Chemical and physical properties of these ligands were obtained consulting the database of odorant responses of The Good Scents Company (http://www.thegoodscentscompany.com/search2.html) and PubChem (https://pubchem.ncbi.nlm.nih.gov/). Part of the ligands were tested also on DsuzOR19As expressed in HEK293T cells (Figure 5) and by GC-SSR on ∆halo *Drosophila melanogaster* expressing DsuzOR19A2 (see supplementary material).

Supplementary GC-SSR experiments were conducted as previously described in the work of Cattaneo et al. (2022). In brief, we interfaced the GC-equipment available in our labs with an SSR rig injecting samples on a 7890 GC System (Agilent technologies Inc., Santa Clara, CA, United States) provided with a 30 m × 0.32 mm fused silica capillary column (Agilent Technologies Inc.), coated with HP-5, df = 0.25 μm, and programmed from 30°C (hold 3 min) at 8°C/min to 250°C (hold 5 min) (software: GC-SSR-1 Agilent, OpenLab, Agilent Technologies). The split of the outlet from the GC column was a 1:1 ratio between the flame ionization detector and the mounted antenna, according to instrument settings. A humidified flow of 3.5–4.0 L/min charcoal-filtered air was directed into a 90-degree-angled glass tube provided with a hole on the angle where the part of the column exiting from the transfer line accessed. Upon conduction of optimization procedures of this method, as described by Cattaneo et al. (2022), recording was re-performed for up to 35 min upon preliminary observation of retention times for the injected compounds.

Using GC-SSR, we tested the insects expressing DsuzOR19A2 subunits (Supplementary Figure S1A) to 10.0 ng aliquots of part of the synthetic ligands indicated in Table 1. Ligands were diluted either in hexane or ethanol depending on the experimental conditions, injecting 2.0 μL into the gas chromatographer. Parallel experiments were conducted testing both the *Haneniaspora uvarum* headspace tested in the work of Cattaneo et al. (2022), which are the headspace collected from apple (Malus LFTA2) and the headspace collected from apple infested with *Haplocampa testudinea* (Haplomalus 564), already available in our labs, that will be part of a different investigation (Cattaneo et al. in preparation). To test headspace collections by GC-SSR, aliquots of 4.0 μL were injected into the gas chromatograph.

### Sequence analyses, prediction of protein topology and structural analysis

Polypeptide sequences of DsuzOR19A1 and DsuzOR19A2 were aligned with Multalin (http://multalin.toulouse.inra.fr/; Corpet (1988)), which unveiled non-conserved amino acids between the two sequences. As carried out in previous studies (Garczynski et al., 2019), transmembrane domains for OR19a proteins were predicted with Topcons (http://topcons.cbr.su.se/; Tsirigos et al. (2015)). Topology for transmembrane domains was predicted using Protter V. 1.0 (http://wlab.ethz.ch/protter/; Omasits et al. (2014)). The results from Multalin and Protter were elaborated using Affinity Designer 1.8.3.641.

The PDB accession from DmelOR19a (UniProt Q9I816) was downloaded from AlphaFold (alphahold.ebi.ac.uk) and submitted to structural analysis using RasTop (https://www.geneinfinity.org/rastop/). This accession was chosen because of the absence of deposited OR19a structures from D*. suzukii* and other species of the genus *Drosophila*. To identify transmembrane domains, a preliminary polypeptide sequence alignment was performed using the ClustalW function of BioEdit (Hall, 1999).

### Heterologous expression in HEK293 cells and transient transfection

Heterologous expressions on human embryonic kidney (HEK293A) cells were conducted following procedures already described in the work of Crava et al. (2022). In brief, HEKs were grown to semi-confluence in 35-mm Petri dishes containing HEK cell media [Dulbecco’s modified Eagle’s medium containing 10% fetal bovine serum (MP Biomedicals, Solon, OH, United States), 2 mM L-glutamine, and 100 mg/mL penicillin/streptomycin (Invitrogen)] at 37°C and 5% CO_2_. Transient expression was conducted co-transfecting 1.2 mg of pcDNA40-DEST-DsuzOR19A1 or pcDNA40-DEST-DsuzOR19A2 with 0.6 mg of pcDNA5/TO (Invitrogen) carrying the CDS of the CpomOrco variant from the codling moth *Cydia pomonella* (GenBank accession number JN836672.1) (Bengtsson et al., 2012). For control experiments, CpomOrco was co-transfected alone. To report expression, 0.6 mg of a separate plasmid DNA carrying the CDS for a blue fluorescent protein (EBFP) was co-transfected [pEBFP2-Nuc, a gift from Robert Campbell, University of Alberta, Alberta, Canada: Ai et al. (2007) (Addgene plasmid #14893)]. Expression of fluorescent reporter genes was under the regulation of the same promoter for Orco/OR genes (CMV). In brief, transfection DNAs were dissolved in 100 mL sterile DMEM mixed with 3 mL CalFectin (SignaGen, Rockville, MD, United States) following the recommended protocol to incubate cells overnight for up to 18 h. After incubation, the HEK cell media were replaced with 2 mL fresh media to incubate the cells at 37°C for up to six to eight additional hours, at which point part of the cell culture was spread in the middle of a 35-mm plate as individual cells or small clusters and rinsed at the sides with 2 mL fresh HEK media. After splitting, the cells were allowed to recover for at least 1 day prior to calcium imaging.

### Imaging experiments on HEK293 cells

Activation of HEK293A cells transfected with CpomOrco, CpomOrco + DsuzOR19A1, and CpomOrco + DsuzOR19A2 was tested using the same procedures we previously described (Cattaneo et al., 2017a; Cattaneo et al., 2017b; Bobkov et al., 2021; Crava et al., 2022). In brief, Petri dishes were incubated for 1 h at room temperature in 1.0 mL HEK Ca^++^Ringer (mM: 140 NaCl, 5 KCl, 2 CaCl_2_, 10 HEPES, pH 7.4) containing the fluorescent calcium indicator Fluo-4 AM (Invitrogen) at 5–15 mM prepared with 0.06%–0.2% Pluronic F-127 (Invitrogen). The buffer was removed after incubation, and cells were rinsed with 4 mL fresh HEK Ca^++^Ringer and placed on the stage of an inverted microscope (Olympus IX-71, Olympus Corp., Tokyo, Japan) equipped with a cooled CCD camera (ORCA R2, Hamamatsu, Hamamatsu City, Japan). The cells were continuously perfused with Ca^++^Ringer using two gravity-fed perfusion contours. The stimulating contour washing of the cells (∼250 mL/min) was switched rapidly to the stimulus contour using a multi-channel rapid solution changer (RSC-160, Bio-Logic, Claix, France) under the software control of Clampex 9 (Molecular Devices, Sunnyvale, CA, United States). Fluorescence imaging was performed using settings optimized for Imaging Workbench 6 software (INDEC BioSystems, Santa Clara, CA, United States) (Cattaneo et al., 2017b). Non-responsive cells were not included in these analyses. Each cell was assigned a region of interest (ROI), and changes in fluorescence intensity within each ROI were measured and expressed as the fractional change in fluorescence intensity (dF). Stored time-series image stacks were analyzed offline using Imaging Workbench 6, Clampfit 10.5 (Molecular Devices LLC, San Jose, CA, United States), and SigmaPlot 11 (Systat Software Inc., San Jose, CA, United States). Amplitudes of the calcium responses to the non-specific Orcoagonist VUAA1 (acetamide, N-(4-ethylphenyl)-2-[[4-ethyl-5-(3-pyridinyl)-4H-1,2,4-triazol-3-yl]thio]-, CAS 525582-84-7, Glixx Laboratories, Southborough, MA, United States) were used to generate dose–response characteristics, and values were normalized to the response amplitude recorded at 1,000 mM of VUAA1. Dose–response curves were approximated using the Hill equation. Constraints were applied in some cases to fit either limited or greatly scattered datasets. Continuous traces of multiple responses were compensated for slow drift of the baseline fluorescence when necessary. All recordings were performed at room temperature (22°C–25°C).

### Stimuli

Compounds tested on human ORs were selected from previous studies demonstrating tuning of OR1A1 and OR2W1 (Adipietro et al., 2012; Geithe et al., 2017) and adding a panel of aldehydes from our previous screening (Cattaneo et al., 2022) that have also been reported to be active on the human receptors (Frey et al., 2022).

Compounds tested on IRs were chosen among amines demonstrated to be active from previous deorphanization findings conducted on the IRs of *D. melanogaster* (Silbering et al., 2011; Hussain et al., 2016; Herre et al., 2022), including possible IR64a activators, which were chosen by consulting Ai et al. (2013) and the Database of Odorant Responses (DoOR) (http://neuro.uni-konstanz.de/DoOR/content/DoOR.php (Munch and Galizia, 2016)).

Most of the compounds tested on DsuzOR19As were selected based on previous findings from the OR19A ortholog of *D. melanogaster* (Dweck et al., 2013).

For experiments conducted on HEK cells, VUAA1 was selected among the ligands that we previously reported active on CpomOrco/OR channels (Bobkov et al., 2021; Cattaneo et al., 2022; Crava et al., 2022). Specifically, we used the same VUAA1 sample adopted for the experiments in the work of Crava et al. (2022), which was dissolved in dimethyl sulfoxide (DMSO, Sigma-Aldrich, St. Louis, MO, United States) and stored as a stock solution (200 mM) at −20°C. The final working concentrations (10–1,000 mM) of VUAA1 were always prepared right before the experiments. Compounds tested on DsuzOR19As were also used for experiments in HEK cells upon the co-expression of DsuzOR subunits with CpomOrco. For these experiments, compounds were diluted in ethanol, as carried out for SSR experiments, and tested at 400 μM in HEK Ca^++^Ringer.

## Results

### Testing the functionality of pOR22a-Gal4

**FIGURE 1:**
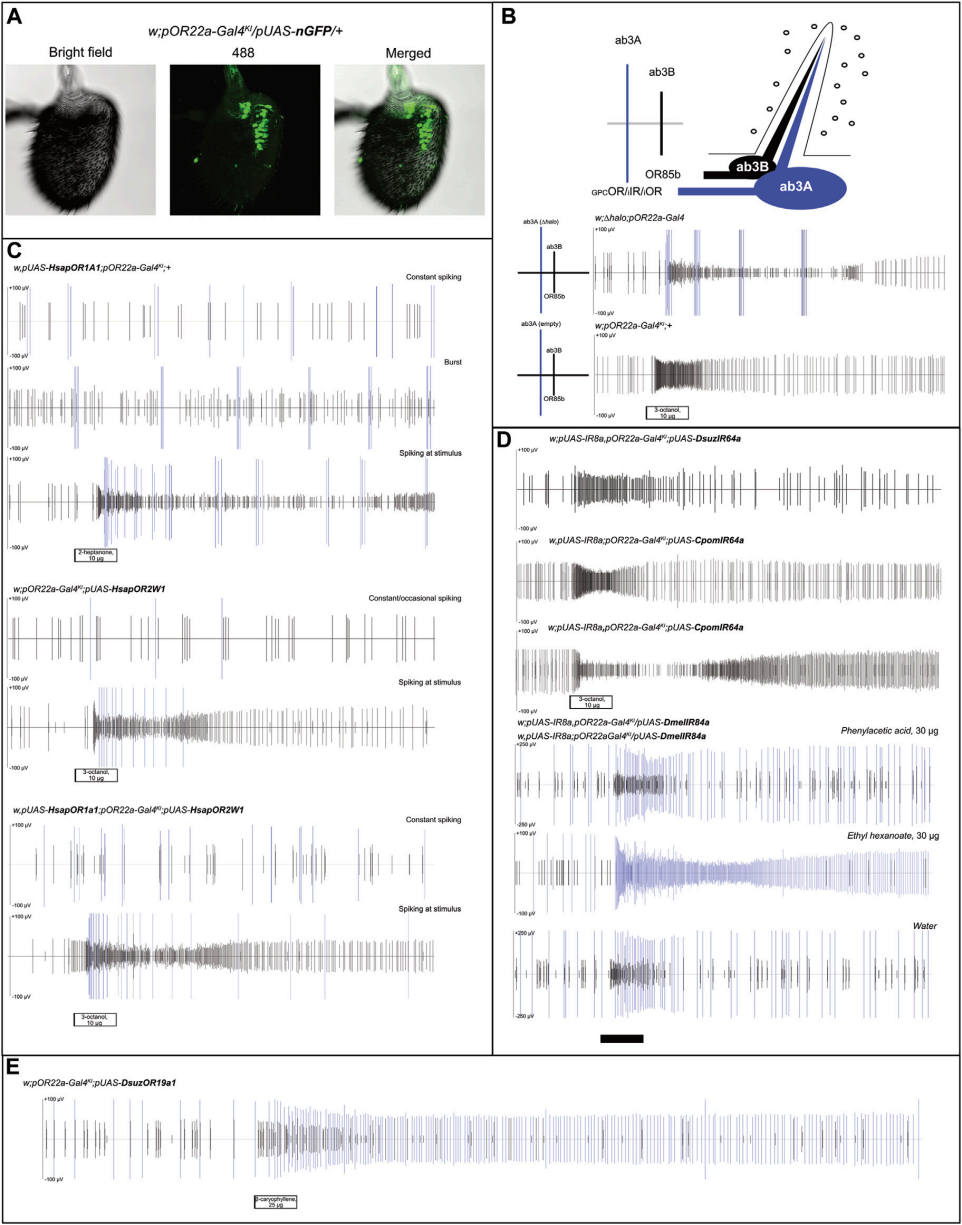
Analysis of ab3A spiking in transgenic *D. melanogaster* expressing human ORs, their combination, and insect IRs. **(A)** Immunostaining testing Gal4/UAS functionality on *Drosophila melanogaster* antennae; nGFP expression in ab3A neurons was obtained upon crossing of *pOR22a-Gal4* ^*KI*^ and *pUAS-nGFP* lines (N = 15), demonstrating effects of Gal4/UAS interaction. **(B)** Small illustration summarizing the status of the ab3 sensillum, with spike scales distinguishing the ab3A spike (“GPC,” *G-protein coupled*; “i,” *ionotropic*) from the ab3B (OR85b). Below: control experiments showing effects of *∆halo* homozygous (*w;∆halo;pOR22a-Gal4* [N = 4] (Cattaneo et al., 2022)) and the Gal4 *knock-in* parental flies (*w;pOR22a-Gal4* ^*KI*^ *;+* [N = 3] (Chahda et al., 2019)); white bar: stimulus. **(C)** Recording from ab3 sensilla showing constant, occasional, or burst-associated ab3A effects depending on the insect tested and the *HsapOR-*transgenesis (N = 4–5). In all cases, we observed ab3A activation associated with ab3B activation to its control ligands (Figure 2A); white bars: stimuli. **(D)** Heterologous expression experiments testing IR expression in ab3A neurons upon crossing with transgenic lines based on the Gal4 insertion into the OR22a/b-locus (*w,pUAS-IR8a,pOR22a-Gal4* ^*KI*^ *;pUAS-DsuzIR64a* [N = 3]; *w,pUAS-IR8a;pOR22a-Gal4* ^*KI*^ *;pUAS-CpomIR64a* [N = 3]; *w,pUAS-IR8a,pOR22a-Gal4* ^*KI*^ *;pUAS-CpomIR64a* [N = 4]; and *pUAS-IR8a,pOR22a-Gal4* ^*KI*^ */pUAS-IR84a;+* [N = 4]). For transgenic lines designed to express IR64a, ab3A spiking was absent. For transgenic lines designed to express IR84a, rather fly lines with *pUAS-IR8a* insertion on the first or on the second chromosomes resulted in the lack of an evident ab3A spiking when compared with water; white and black bars: stimuli. **(E)** Tonic effect of ab3A spiking expressing DsuzOR19A1 when tested with 25 μg of β-caryophyllene (replicate 2, Supplementary Data File S1); black bar: stimulus.

Before starting crossing to obtain transgenic lines for SSR experiments, we assessed the functionality of the *pOR22a-Gal4* ^*KI*^ constructs (Chahda et al., 2019). Antennal immunostaining of insects, whose Gal4 expression was designed through this system, resulted in an evident nGFP expression in neurons located in proximity of the antennal region I (Figure 1A) including ab3s and other large basiconic sensilla (de Bruyne et al., 2001; Dobritsa et al., 2003).

When performing SSR on the ab3 sensilla of parental *pOR22a-Gal4* ^*KI*^ lines, contrary to our previous findings when testing *∆halo-*empty neuron flies (Cattaneo et al., 2022), we observed a lack of ab3A burst even at high doses of ab3B activators (3-octanol 10 μg, Figure 1B), which is commonly known as a typical effect of *∆halo* (Dobritsa et al., 2003).

### Heterologous expression of human GPCRs in ab3A neurons of *Drosophila melanogaster*

Replacing the expression of single human GPCRs, OR1A1, and OR2W1, or their co-expression into the ab3A neurons (Figure 1C), displayed basic spiking, where the phenotype and frequency differed depending on the replicate. This showed constant spiking (OR1A1 or OR1A1+OR2W1) or burst phenotype (OR1A1). The ab3A burst is a common abnormality in the phenotype of this neuron that was observed for the first time in *∆halo* mutants, whose activity was influenced by the cactivation of ab3B and rescued by expressing OR22a (Dobritsa et al., 2003). Such an effect was not described in CRISPR-gene-edited empty ab3A flies (Chahda et al., 2019), and in accordance, we did not observe this phenotype when we tested these fly lines (Figure 1B). Although in our recordings, we observed that this burst can also be induced by ab3B activation (Figure 1C), evidence from its absence in the *pOR22a-Gal4* ^*KI*^ lines (Figure 1B) suggests it to be possibly associated with GPCR expression.

**FIGURE 2:**
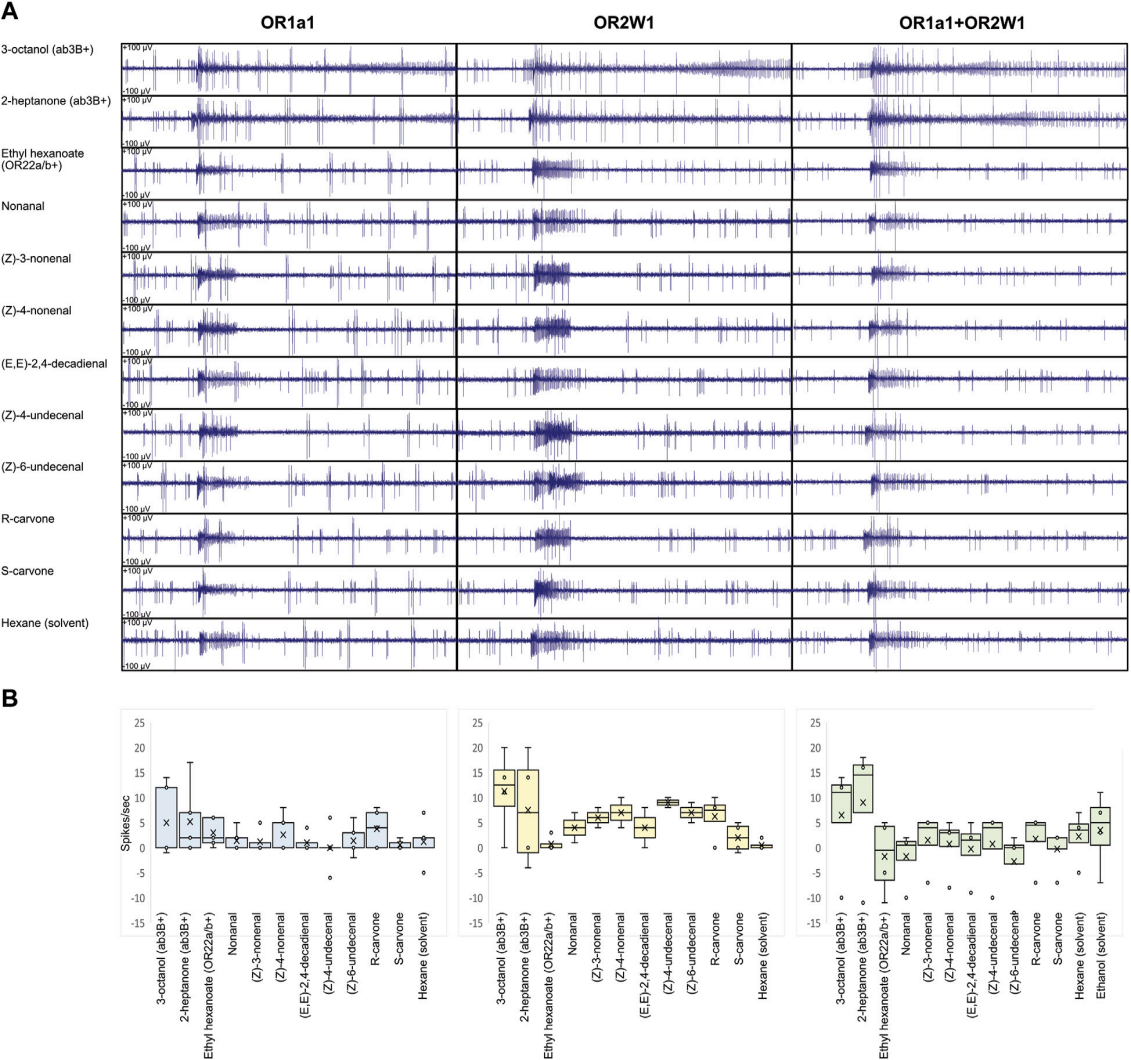
Ligand screening on transgenic *D. melanogaster* expressing human ORs and their combination in ab3A neurons. **(A)** Ligand screening showing lack of evident ab3A effect on the tasted compounds from Table 1 (N = 2–5). The effect of ab3B conserved when puffing the solvent suggests it to be associated with an artifact. **(B)** Respective box plot analysis of ab3A spiking, indicating HsapOR1A1 in light blue, HsapOR2W1 in light yellow, and HsapOR1A1+OR2W1 in light green. No significant differences were identified when ab3A effects were compared with the respective solvents (N = 2-5; *p* > 0.05; Supplementary Data File S1).

For all the tested genotypes, when applying different ligands, we observed cases where spiking was limited only at the stimulus; this was evident when we used ab3B activators, such as 2-heptanone and 3-octanol (Figure 1C, Figure 2A, Supplementary Data File S1). Although this effect may suggest a possible activation of the GPCR-ORs that we have heterologously expressed, its inconsistency among the various replicates and evidence of ab3A spiking even when applying solvents excluded such an effect as a possible response to ligands (Figure 2), where ab3A spiking was non-significant when compared with the solvent (Figure 2B, Supplementary Data File S1).

### Heterologous expression of insect IRs in ab3A neurons of *Drosophila melanogaster*

In a parallel set of experiments, we used the same method to test transgenic insects designed to express into ab3As IR transgenes from *D. suzukii* (DsuzIR64a) and *C. pomonella* (CpomIR64a) (Cattaneo et al., 2023), as well as *D. melanogaster* IR84a (Abuin et al., 2011) (Figure 1D). Although we tested two genotypes based on differing *pUAS-IR8a* insertion on chromosomes I or II (Supplementary Figure S1), we did not observe ab3A spiking to associate with a possible activation of IR8a-co-expressed IR64a subunits. Testing a panel of ligands including acids and amines, known to be among the main IR activators (Table 1), unveiled no responses (Supplementary Figure S2).

When we attempted to express IR84a into ab3A neurons, we did not observe any spiking from puffing phenylacetic acid; instead, we observed a basic ab3A spiking related with OR22a/b, which was demonstrated by the effect on ethyl hexanoate. For our replicates, we used heterozygous genotypes in which the OR22a/b locus was still present in one copy of chromosome II (*pOR22a-Gal4* ^*KI*^ */pUAS-DmelIR84a*).

### Heterologous expression of insect ORs in ab3A neurons of *Drosophila melanogaster*

**FIGURE 3:**
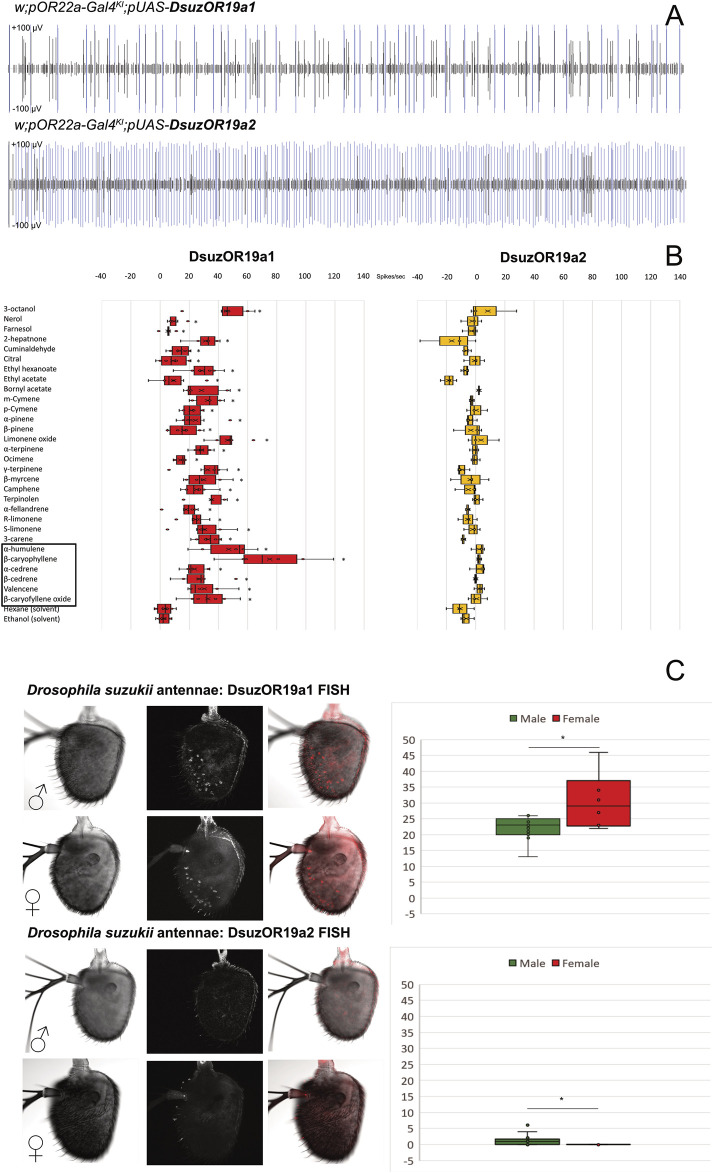
Functional characterization and expression analysis of DsuzORs. **(A)** Spike-train comparison of ab3A neurons expressing DsuzOR19A1 (above) and DsuzOR19A2. In all of our experiments, we observed higher firing rate for OR19A2. **(B)** Odorant response profile of ab3A neurons from transgenic *Drosophila melanogaster* expressing OR19A1 and OR19A2 subunits, tested with the compounds reported in Table 1. Asterisks depict compounds enhancing significant spiking for OR19A1 (N = 6; *p* < 0.05; Supplementary Data File S1). Black square depicts sesquiterpenes enhancing tonic effect. **(C)** FISH expression analysis comparing *Drosophila suzukii* antennal samples collected from males and females between OR19A1 (N_males_ = 13; N_females_ = 6; above) and OR19A2 (N_males_ = 16; N_females_ = 13; below). Right: box plots derived from neuronal counting; asterisk indicates a significant difference in the number of neurons between males and females, for both OR19A1 (*t*-test: *p* = 0.03394; ɑ = 0.05) and OR19A2 (MWU: *p* = 0.004; z = −3.382; U = 39; ɑ = 0.05). Expression of OR19A2 is limited to the sole male antenna, and except in few cases, fluorescent staining is limited to only one neuron (Supplementary Data File S2).

The heterologous expression of *D. suzukii* ORs in *D. melanogaster* unveiled a clear ab3A spiking with different frequencies between OR19A1 and OR19A2 (Figure 1E, Figure 3A). Recovering different rates of ab3A spiking in the progenies generated from crossings of parental *pOR22a-Gal4* ^*KI*^ lines with *w;Bl/Cyo;pUAS-DsuzOR19A* mutants (Supplementary Figure S1A) confirmed the expression of both the *DsuzOR19A* transgenes (Figure 1B, Figure 3A), which is in accordance with our previous findings testing other OR subunits of *D. suzukii* (Cattaneo et al., 2022). Testing a panel of ligands that we selected based on the previously investigated orthologs from *D. melanogaster* (Dweck et al., 2013) (Table 1), we observed that DsuOR19A1 significantly responded to all of these compounds but showed reduced magnitudes for the effects of nerol (*p* = 0.027), farnesol (*p* = 0.046), citral (*p* = 0.046), and ethyl acetate (*p* = 0.028) (Figure 3B, Supplementary Data File S1). Interestingly, we observed effects ranging from highly phasic to highly tonic (Supplementary Figure S3). In particular, tonic effects were specific for cyclic sesquiterpenes (Table 1, Figure 3B, and Supplementary Data File S1). Contrary to DsuzOR19A1, compounds tested on DsuzOR19A2 unveiled no effects (Figure 3B). Additional studies on transgenic flies expressing DsuzOR19A2 in the *∆halo-*empty neuron system, by injecting the equipment we optimized to perform GC-SSR (Cattaneo et al., 2022) (Supplementary Figure S4), confirmed both the recovering rate of ab3A spiking associated with this subunit and its absence of activation of most of the ligands that we tested, even at high dosages (10.0 ng).

### Expression analysis of DsuzOR19A subunits in *D. suzukii* antennae

Fluorescent *in situ* hybridization analysis unveiled evident expression of DsuzOR19A1 in both male and female antennae. Testing normality for neuronal counts for DsuzOR19A1 unveiled data being normally distributed (Shapiro–Wilk: *p* = 0.093), contrary to the neuronal count for DsuzOR19A2 (Shapiro–Wilk: *p* < 0.001). Testing variances between the two samples of male and female antennae for DsuzOR19A1 unveiled a significant difference between the neuronal counts from the two genders [F (1.17) = 4,997; *p* = 0.039; Supplementary Data File S2]; a one-tailed two-sample heteroscedastic *t*-test unveiled a significantly higher number of neurons for females (30.50 ± 8.87) compared to males (22.08 ± 3.62) (*p* = 0.03394; ɑ = 0.05).

When using probes for DsuzOR19A2, very few neurons were stained in males (1.50 ± 2.03), and no neurons were stained in females (Figure 3C). The Mann–Whitney *U*-test unveiled a significant difference between these data (*p* = 0.004; z = −3.382; U = 39).

Based on their positioning (Figure 3C), comparison from recent deposited findings mapping sensilla from *D. suzukii* antennae (Keesey et al., 2019) would suggest our neurons housing into either ab8-ab10, ai2/ai3, or at1/at4 sensilla.

Despite very similar results, we observed slight morphological differences between the antennae of *D. suzukii* and *D. melanogaster* (Figure 1A), as unveiled by a parallel set of experiments that we performed in the phase of optimization of the *in situ* protocol using DsuzOrco positive control probes (Supplementary Figure S5).

### Sequence and tridimensional analysis of the DsuzOR19A subunits

**FIGURE 4:**
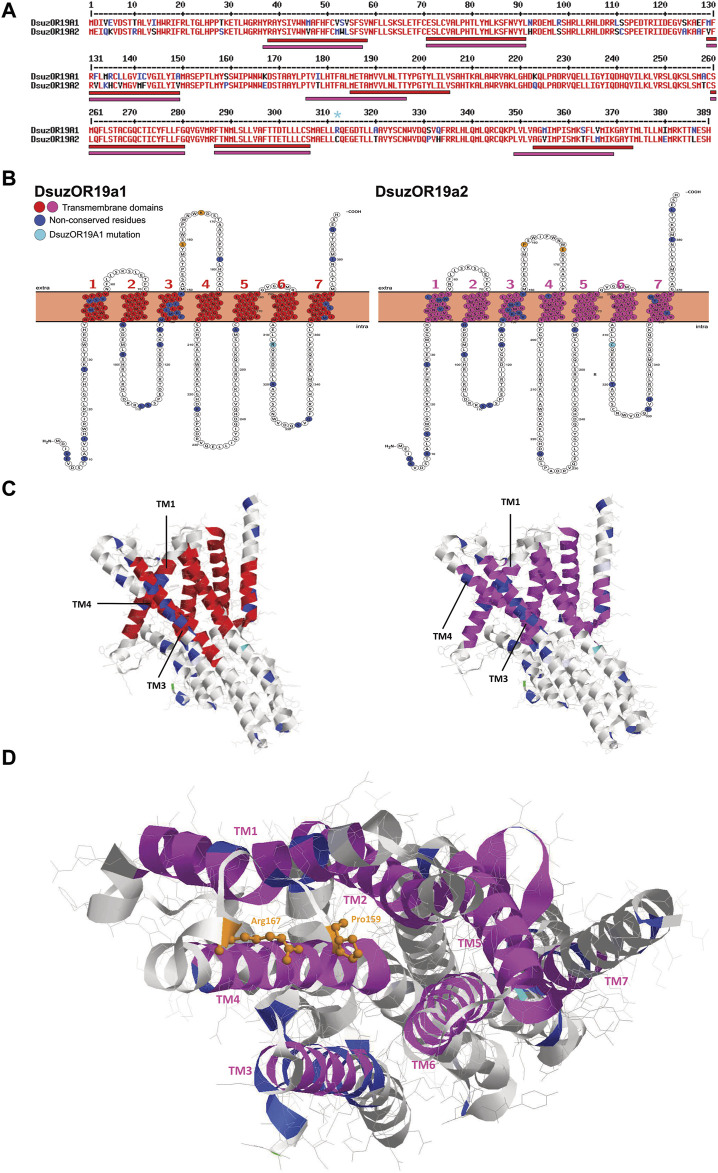
Sequence, structural, and tridimensional analysis of DsuzORs. **(A)** Polypeptide sequence alignment showing non-conserved amino acid residues between OR19A1 and OR19A2. Light blue asterisk denotes the OR19A1 mutation that we identified in our sample. Bars indicate transmembrane domains of DsuzOR19A1, red, and DsuzOR19A2, magenta. **(B)** Transmembrane topology of DsuzOR19A1 and DsuzOR19A2 showing transmembrane colors as in **A**, while non-conserved amino acid residues are colored blue. Non-conserved amino acid residues from the extracellular loop 2 are shown in orange; the extra amino acid substitution within the ICL-3 (Arg312) that we identified in our samples compared with sequences deposited by Ramasamy et al. (2016) is indicated in light blue. **(C)** Tridimensional structure of DsuzOR19A subunits based on the UniProt Q9I816 PDB model from OR19a of *Drosophila melanogaster*. Structures were oriented by putting the TM7-C-terminal straight on the right. Note: colors were adopted as in **C**; methionine 1 is indicated in green. **(D)** Extracellular pocket formed by the loose packing of TM1–TM6 helixes, showing in orange the amino acids Pro159 and Arg167 based on DmelOR19a laying within the pocket, expected to be substituted to serine and arginine in DsuzOR19A1 and proline and glutamate in DsuOR19A2.

Polypeptide sequence alignment of DsuzOR19A1 and DsuzOR19A2 unveiled several non-conserved amino acid substitutions (Figure 4A). Snake-plot membrane topology resulted in a more or less homogeneous distribution of these substitutions among the various intracellular, extracellular, and transmembrane domains. Analyzing the transmembrane (TM) domains, TM1 and TM3 locate most of the amino acid substitutions, with TM1 positions differing for one amino acid shift (TM1_DsuzOR19A1_: 38-58; TM1_DsuzOR19A2_: 37–57). While TM2, 5, and 6 are conserved between the two subunits, both in terms of positions and amino acid sequences, TM4 and TM7 differ in their organization and amino acid composition, resulting in a shorter extracellular loop-2 and a longer C-terminal for DsuzOR19A2 (Figure 4B).

Tridimensional analysis of DsuzOR19A1 and DsuzOR19A2 based on the UniProt Q9I816 PDB model from the OR19a ortholog of *D. melanogaster* unveiled TM1 and TM3 being spaced by TM4 (Figures 4C,D), above which two substitutions in the extracellular loop 2 extend within the extracellular pocket formed by the loose packing of helices TM1–TM6 (Figure 4D).

Interestingly, taking the deposited OR19A1 and OR19A2 as a reference (Ramasamy et al., 2016), our sample OR19A1 is provided with an extra amino acid substitution within the ICL-3 (Arg312) (Figures 4A–D).

### Heterologous expression of insect ORs in HEK293T cells

**FIGURE 5:**
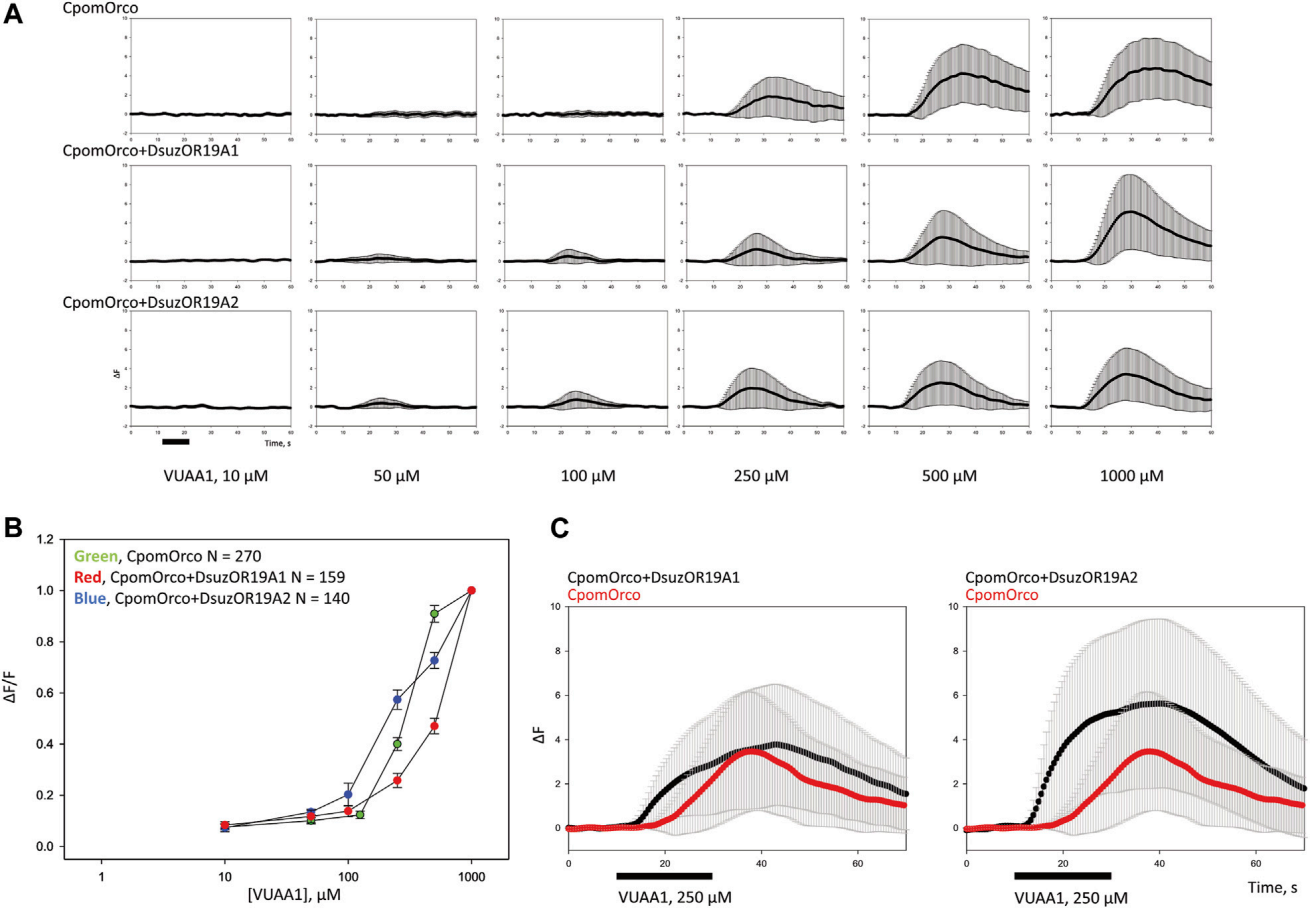
Heterologous expression of DsuzOR19A1 and DsuzOR19A2 in HEK293T cells. **(A)** VUAA1 stimulation elicits a dose-dependent Ca^++^_i_ increase in HEK293T cells expressing either homomeric CpomOrco [N = 270] or chimeric CpomOrco + DsuzOR complexes (DsuzOR19A1 [N = 159], DsuzOR19A2 [N = 140]), following similar methods described by Crava et al. (2022). Data within each row were obtained from single preparation; panels indicate the respective concentration dependences. The maximal agonist concentration used in these experiments (VUAA1 1,000 μM) is likely not a saturating concentration, as previously demonstrated (Cattaneo et al., 2017b; Bobkov et al., 2021). Black bar: stimulus. **(B)** Concentration dependences of VUAA1. Data were obtained in the separate series of experiments. The response amplitudes were used to generate the agonist concentration dependences. The average peak amplitudes of the responses of different cells (N = 140–270) were normalized to the maximal responses usually elicited by the application of a saturating concentration (1,000 μM) of VUAA1. **(C)** Comparison of the effect to VUAA1 between different cell samples expressing rather CpomOrco homomers [N = 73] or CpomOrco + DsuzOR19A heteromers (DsuzOR19A1 [N = 94], DsuzOR19A2 [N = 96]). Black bar: stimulus, VUAA1 = 250 μM.

Comparing cell samples expressing homomeric CpomOrco with chimeras co-expressing CpomOrco + DsuzORs, we observed responses to the Orco VUAA1-agonist at lower dosages for the chimeric channels (Figure 5A). We did not observe convincing differences in agonist sensitivity between homomeric CpomOrco ([VUAA1]1/2–284 ± 39 μM) and its chimeric co-expression with DsuzOR19A2 ([VUAA1]1/2–389 ± 219 μM), while the trend of the dose–response curve for DsuzOR19A1 resulted in a [VUAA1]1/2 overestimation (∼5.0 E+07 μM) (Figure 5B). However, some experiments demonstrated an overall response to VUAA1 being more tonic than CpomOrco alone when cells were stimulated with high dosages of this agonist (Figure 5C).

Contrary to our expectation, ligands from our screening (Table 1) that activated DsuzOR19A1 when tested by SSR (Figure 3B) did not result in any observable fluorescence variation when perfused on these cells.

## Discussion

In this work, we attempted the heterologous expression of both GPCRs and ligand-gated cation channels into ab3A neurons of transgenic *D. melanogaster* by the use of a designed knock-in line replacing the OR22a/b locus with Gal4 (Chahda et al., 2019). While our attempts of the expression of GPCR subunits in these neurons re-established ab3 spiking as an evidence of a possible cation influx (Figure 1, Dobritsa et al. (2003)), when we tested the main ligands for human OR1A1 and OR2W1, we observed no effects (Figure 2). Among the various non-GPCR insect subunits that we attempted to express into the ab3A neurons (Figures 1–3), only the *D. suzukii* subunit OR19A1 resulted being functional (Figure 3). By conducting screenings of various ligands that we selected among the most active for the OR19a ortholog of *D. melanogaster* (Dweck et al., 2013), we demonstrated the activation of DsuzOR19A1 to all these ligands. Although the expression into the ab3As of *D. suzukii* OR19A2 unveiled evident spiking, as an evidence of its confirmed heterologous expression (Figure 3), the ligand screening on this OR19A resulted in lack of activation.

Evidence of limited ab3A spiking when we attempted to express GCPRs into ab3A neurons (Figure 1) directed our efforts towards selecting only the most active ligands for these ORs, based on studies reported for OR1A1 (Geithe et al., 2017) and OR2W1 (Adipietro et al., 2012), indicating both the isomers of carvone as the most promising (Figure 2). In addition, we included several aldehydes in our screening, among which (Z)-4-nonenal, (Z)-4-undecenal, and (Z)-6-undecenal have been more recently demonstrated to be the strongest candidates to activate OR2W1 (Frey et al., 2022). While the given absence of any effect may indicate the basic ab3A spiking to be an artifact (Figure 2), we cannot exclude that such spiking may result from the expression of a metabotropically regulating subunit involved in gating the wide asset of ion channels that are expressed in *Drosophila* neurons (Hodge, 2009). On the other hand, such spiking phenotype might be related to changes in Ca^++^ homeostasis due to the stress of the endoplasmic reticulum (Groenendyk et al., 2021). To test this, additional studies may investigate subcellular localization of the transgenic receptors in the endomembrane systems.

Since long ago, the expression of GPCRs for pharmacological purposes has been known in all major heterologous systems (Tate and Grisshammer, 1996), including insect cells, in which cases of expression of non-functional forms of GPCR receptors have been reported (Parker et al., 1991). Although insect cells in general, and *Drosophila* in particular, do not lack G-proteins in their sensory neurons (Kain et al., 2008; Yao and Carlson, 2010; Deng et al., 2011), it is unclear whether the non-functionality of heterologous GPCRs may be caused by additional factors, such as lack of glycosylation or possible impairments in protein folding. Furthermore, the overall low abundance of cholesterol in insect cells is known to alter GPCR ligand-binding capacities, as was reported in the case of the oxytocin receptor (Gimpl et al., 1995). Although possibly, all these reasons may justify the absence of response to the ligands from Table 1 that we observed when we tested OR1A1, OR2W1, and their combination (Figure 2).

For every transgenic fly line expressing IRs that we tested, we observed an absence of ab3A spiking (Supplementary Figure S2), as was the case for the negative control *w;pOR22a-Gal4* ^*KI*^ *;+* (Figure 1B), suggesting a possible lack of any IR expression in our ab3A system. In addition, contrary to the findings of Abuin et al. (2011), our misexpression attempts for IR84a into ab3As did not result in any effect to its main ligand phenylacetic acid (Silbering et al., 2011) (Figure 1D). Up to our knowledge, the study by Abuin et al. (2011) is the sole one that adopted ab3A to express IRs. Contrary to the transgenic lines we used (see Materials and methods), the lines adopted in this study to perform crossings for SSR were provided with an insertion including an EGFP protein fused with the IR-CDS (*UAS-EGFP:IR84a*) and with a *pOR22a-Gal4* insertion on the III chromosome (Vosshall et al., 2000). Instead, the *pUAS-IR8a* line used in both our study and the study by Abuin et al. (2011) was the same. We do not know if adopting a specific parental Gal4 line rather than another may improve or compromise a successful pUAS expression (Dobritsa et al., 2003; Chahda et al., 2019). In any case, the study by Abuin et al. (2011) demonstrated an efficient neuronal staining when testing IR84a expression *in situ*.

Interestingly, the IR84a polypeptide sequence from the work of Abuin et al. (2011) (GenBank HQ600591.1) presented two main amino acid substitutions (W11L and F206L) when compared with the polypeptide sequence of the IR84a inserted in the parental flies that we used (BDSC#41740). Apart from this, tryptophan or phenylalanine rather than leucine do not expect critical differences in physicochemical properties, and occurring within positions at the N-terminal preceding the S1 (Benton et al., 2009), they are not expected to affect functionalities from the ligand-binding domain of the IR84a channel.

In the last decade, experiments conducted *in situ* on antennal neurons of *D. melanogaster* unveiled OBP19a among the main binding proteins that are specific for neurons of basiconic sensilla, including the ab3s (Larter et al., 2016). We do not know whether the eventual lack of binding capacities for variants of this protein to phenylacetic acid may justify the lack of activation for the IR84a subunits that we misexpressed (Figure 1D). Despite this not being part of our targets, further trials may investigate upstream binding capacities for phenylacetic acid or (eventually) other ligands of Table 1 with DmelOBP19a upon its expression and purification *in vitro* (Zhang et al., 2022; Jia et al., 2023), with the intent to unveil possible interference upstream the IR84a receptor activation.

We still do not know why our attempts to express IRs into ab3A neurons did not work. While future efforts may attempt functional expression of other IRs, we are aware that further *in situ* investigations are needed to demonstrate the expression and targeting of the ab3A dendrites of the IR subunits that we tested, including IR84a. The possibility remains that our transgenic flies might not carry the expected transgenes and/or that the transgenes are not expressed as expected. Additional RT–PCR analysis on the antennae of *D. melanogaster* IR84a-knockouts, cis-expressing IR84a within ab3A neurons, may shed further light confirming if this IR is expressed or not. Further experiments may add to our results, as mentioned above, but there are no additional reports apart from the work of Abuin et al. (2011) attempting IR cis or heterologous IR expression into ab3A neurons, although IR co-receptors are co-expressed with ORs in the *D. melanogaster* ab3As (Vulpe and Menuz, 2021; Menuz, 2022; Task et al., 2022). Together with this lack of reports, although speculative, our results seem to suggest IRs not functioning into ab3A neurons due to the absence of important co-factors.

Contrary to human GPCRs and insect IRs, the expression of one out of the two insect ORs that we have chosen unveiled a response to various ligands (Figure 3B). All ligands activated DsuzOR19A1 (Supplementary Data File S1). According to previous studies conducted on orthologs from *D. melanogaster* (Dweck et al., 2013), nerol, farnesol, and citral were among the less active. In addition, we recorded effects on ethyl acetate, known for being active on *D. melanogaster* neurons housing in ab1 and ab2 sensilla, which are located on the same antennal side of ab3 sensilla (de Bruyne et al., 2001). As in the work of Dweck et al. (2013), we reported the highest effects for β-caryophyllene and the lowest for nerol. Instead, contrary to their studies, it seems that the OR19A1 of *D. suzukii* associates a wider activation spectrum, which, apart from terpens, also spreads among alcohols (3-octanol), ketones (2-hepatnone), aldehydes (cuminaldehyde), and esters (ethyl hexanoate, ethyl acetate, and bornyl acetate) (Figure 3B, Supplementary Data File S1).

Interestingly, the overall responses to the ligands that we tested unveiled effects ranging from highly phasic to highly tonic (Supplementary Figure S3), with tonic effects specific for cyclic sesquiterpenes (Table 1, Figure 3B, and Supplementary Data File S1). In their study, Dweck et al. (2013) did not distinguish whether the OR19a effects on ligands were phasic or tonic; however, like in the work of Dweck et al. (2013), our deorphanization of *D. suzukii* OR19A1 unveiled that apart from sesquiterpenes, the monoterpene limonene oxide resulted among the most active ligands. In terms of the difference between phasic or tonic firing rates, previous studies hypothesized that one effect or the other may be associated to odorant stimuli providing different types of information about the source or the environment from which they are emitted (Hull and Cribb, 2001). However, we do not know if the observed differences in temporal firing pattern for DsuzOR19A1 to sesquiterpenes rather than to other ligands may reflect any sort of behavioral response from *D. suzukii* flies, as it was observed from other dipterans that are phylogenetically close to this insect (Olsson et al., 2006a; Olsson et al., 2006b). Behavioral studies on *D. suzukii* may shed light on possible dimensions behind deciphering the tonic signal from sesquiterpenes compared with the other compounds that we tested, including the ones enhancing strong phasic effects such as limonene oxide (46.33 ± 4.65 spikes/sec).

Another possible scenario is that tonic rather than phasic responses could be the result of a lack of odorant-degrading enzymes in the ab3 sensillar lymph of *D. melanogaster*. In general, when heterologously expressed in the *Drosophila* empty neurons, ORs may maintain a signal termination similar to the one in their native ORN, playing a key role in signal dynamics (Hallem et al., 2004). However, several mechanisms may contribute to signal termination, among which odorant-degrading enzymes are known to rapidly inactivate odorants in the vicinity of the sensory receptors (Chertemps et al., 2012), which is an evidence that the cellular environment could also play a role in the dynamics of the OR response. In support of this hypothesis, previous studies reported rapid termination of response from an OR of the silk moth *Bombyx mori* expressed in *Drosophila* at1 sensilla that was delayed when the same receptor was expressed in another type of sensilla than the at1s (Syed et al., 2006). To validate whether this may be the case of our experiments, and if tonic rather than phasic effects may result from the lack of an appropriate asset of odorant-degrading enzymes, future recordings *in vivo* on *D. suzukii* antennae have to be compared with our heterologous findings. To this target, our *in situ* hybridization results (Figure 3C) may represent a starting point for the detection of sensilla expressing the DsuzOR19A1 subunit on the antennae of *D. suzukii*.

Contrary to DsuzOR19A1, we were not able to observe effects when we tested transgenic *D. melanogaster* expressing DsuzOR19A2 (Figure 3B, Supplementary Figure S4, Supplementary Data File S1). However, evident ab3A spiking from this subunit when it was heterologously expressed may indicate its forming of functional cation channels (Figure 3A, Supplementary Figure S4A). In addition, the limited number of neurons that were identified only from male antennae (Figure 3C) may indicate possible evidence of sexual specificity for DsuzOR19A2 or, still possibly, a very specific tuning to precise ligands, requiring a wider asset of compounds to be tested in future deorphanization efforts. Analyzing the polypeptide sequence of DsuzOR19A1 and DsuzOR19A2 and their snake-plot transmembrane topology (Figures 4A,B), we noticed several amino acid substitutions diffused along the whole sequences, particularly within TMs 1 and 3. Transmembrane prediction unveiled alternative organizations for transmembranes 1, 4, and 7, which for DsuzOR19A2 resulted in shortening of the N-terminal domain and the extracellular loop 2 and an elongation of the C-terminal. While differences in the polypeptide sequences or in the topological organization of the TMs and of the intra/extracellular domains are generally considered at the base of altered binding capacities for OR receptors (Miller and Tu, 2008; Turner et al., 2014), more evidence to explain lack of tuning for DsuzOR19A2 may raise from its 3D analysis (Figure 4D). Comparing DsuzOR19A1 and DsuzOR19A2, we observed two among the non-conserved residues (S159P and K167E) extending within the extracellular pocket formed by the loose packing of helices TM1–TM6, where the 3D modeling of insect OR channels (Butterwick et al., 2018) described it as a potential site for odorant binding. Interestingly, residues from this pocket have been previously indicated in defining odorant specificity among various insect ORs (Nichols and Luetje, 2010; Leary et al., 2012; Hughes et al., 2014; Yang et al., 2017). Despite this, the extensive sequence diversity of ORs may reflect the existence of additional odorant-binding sites distributed throughout these proteins that broaden a receptor’s tuning capacity (Butterwick et al., 2018).

Studies that are more recent investigated 3D structures of the OR5 from the bristletail *Machilis hrabei* (Archaeognatha: Machilidae), identifying the S2, S3, S4, and S6 transmembrane helices splaying apart to form a 15 Å-deep ligand-binding pocket within the extracellular leaflet of the bilayer (del Mármol et al., 2021). This pocket is enclosed within a hydrophobic box constructed from ten large aromatic and hydrophobic residues: Val88, Tyr91, Phe92, Ser151, Gly154, Trp158, Met209, Ile213, Tyr380, and Tyr383. Aligning the polypeptide sequences of *M. hrabei* OR5a (PDB: 7LIC_A) and DsuzOR19As, we identified that seven out of these ten residues have corresponding residues that are identical between both the DsuzOR19As, among which one is conserved with *M*. *hrabei* OR5 (Tyr91) and three are substituted with residues having similar physicochemical properties (Val88Leu, Ile213Val, and Tyr380Phe; Supplementary Figure S6A). By observing that the *M. hrabei* OR5a has predicted transmembrane domains that are about twice longer than the DsuzOR19A-TMs (Figure 4A, Supplementary Figure S6A), it is difficult to derive any conclusion from this finding. However, 3D modeling indicates that some of these seven residues extend their lateral chains within a pocket formed by TM2, TM4, and TM6 (Supplementary Figure S6B), but TM3 takes no part in the formation of this pocket. In addition, the non-conserved residue at position 159 from the ECL-2 (Figure 4C) extends within this TM2–TM4–TM6 pocket suggesting, although hypothetically, a possible continuation of the extracellular TM1–TM6 pocket (Butterwick et al., 2018) within this candidate ligand-binding pocket (del Mármol et al., 2021).

Despite structural/3D analysis not being part of our study, the existence of different residues within a possible extracellular pocket for DsuzOR19A2, extending within a possible ligand-binding pocket with conserved residues, and differences within the polypeptide sequences or in the topological organizations of the DsuzOR19A subunits (Figure 4) may justify, all together, our evidence of absent tuning capacities for DsuzOR19A2.

In a different scenario, DsuzOR19A2 may deserve further functional characterization efforts by performing its heterologous expression into tricoid at1a OSNs. In *D. melanogaster*, OR19a/b are expressed into at3 neurons (Couto et al., 2005), which is an evidence of the possibility that tricoid sensilla may provide a more suitable extracellular environment for OR19a receptor functionalities. In addition, although the fluorescent analysis we conducted *in situ* on *D. suzukii* antennae did not unveil clear sensillar morphologies, evidence from the expression of the OR19A2 subunit in a limited number of neurons identified in the sole males (Figure 3C) may suggest a possible role of this subunit as a pheromone receptor. Pheromone receptors may respond more efficiently when expressed in at1a OSNs rather than ab3A (Syed et al., 2010; Montagne et al., 2012), as we have already demonstrated by comparing functional studies of the *C. pomonella* OR3 into both ab3A and at1a neurons (Bengtsson et al., 2014; Cattaneo et al., 2017a). From these pieces of evidence, future projects may investigate the functional properties of DsuzOR19A2 expressed in at1a OSNs. To these attempts, *D. suzukii* cuticular hydrocarbons (Snellings et al., 2018; Wang et al., 2020), headspace collections (Kwadha et al., 2021; Cattaneo et al., 2022), or candidate sex pheromones, such as (Z)-7-tricosene (Zhan et al., 2021) or (Z)-9-tricosene (Lima et al., 2023), may be tested on this subunit in search of its main ligands.

In a parallel set of experiments, we attempted co-expressing DsuzOR19As with CpomOrco in HEK293T cells. The choice of CpomOrco was based on the conservation of the Orco subunit within various insect orders (Butterwick et al., 2018) and from the functional evidence of the heterologous expression of ORs from various insects within the empty neurons of *D. melanogaster*, forming functional cation channels with DmelOrco (Larsson et al., 2004; Gonzalez et al., 2016). For these reasons, we did not consider possible issues to re-propose the same approach *in vitro*, attempting this time the use of CpomOrco to test ORs from *D. suzukii* in a similar way that ORs from *Papilio* have been studied by Crava et al. (2022). Comparing cell samples expressing homomeric CpomOrco with chimeras co-expressing CpomOrco + DsuzORs, we observed responses to the Orco agonist VUAA1 at lower dosages (VUAA1 = [50-100] μM) for chimeric co-expression (Figure 5A). Although the trend of the dose–response curves and their estimated EC50 were not convincing (Figure 5B), phasic effects when HEK cells co-expressed CpomOrco + DsuzOR19As chimeras suggested that (although possibly) the expression of OR19A subunits of *D. suzukii* in HEK cells was functional (Figure 5C). Despite this, we did not observe any fluorescence variation to the ligands that we found active by SSR when they were perfused on the cell system (Table 1). In our previous studies, the co-expression of CpomOrco with its respective ORs from *C. pomonella* (Cattaneo et al., 2017b; Bobkov et al., 2021) or with ORs from other lepidopterans (Crava et al., 2022) provided evidence of functionalities and led to the CpomORs deorphanization. Despite the suggestion of CpomOrco as a functional co-receptor when used in HEK293T cells, we recognize that it may be inadequate when performing functional studies of ORs from insects not belonging to Lepidoptera, such as *D. suzukii*. Further attempts are deserved for co-expressing DsuzORs with DsuzOrco to test *in vitro* responses to the ligand that we found active from the SSR experiments. On the other side, it is also possible that ORs from *D. suzukii* would be more easily heterologously expressed into neurons of *D. melanogaster*, forming functional cation channels with the native DmelOrco (Larsson et al., 2004), as expected from the phylogenetic proximity and similarities of the chemosensory subunits of these two *Drosophila* (Ramasamy et al., 2016; Walker et al., 2023). In parallel, it is also possible that for specific ORs such as DsuzOR19As, the vapor-phase odor delivery in the empty neuron technique would provide a more realistic physicochemical environment compared to the water-phase odor delivery by an HEK-based system (Gonzalez et al., 2016; Cattaneo, 2018).

Since the first documentations of OR19 subunits among the asset of the ORs expressed by *D. melanogaster* (Warr et al., 2000), OR19a and OR19b have been identified in tricoid at3 neurons projecting to the dorsal/medial DC1 glomerulus (Couto et al., 2005). Neurons expressing these subunits have been found modulating sensing of alkanes, alcohols, ketones, esters, and terpenoids (Fishilevich and Vosshall, 2005), as confirmed by meticulous studies performed through extracellular single-unit recordings (Hallem and Carlson, 2006). Apart from receptors’ expression and their neuronal projections, transcriptomic studies added to the OR19a deorphanization by testing its odorant-dependent alteration of mRNA levels by an approach known as the *high-throughput deorphanization for chemosensory receptors* (Koerte et al., 2018). Apart from these studies, Dweck et al. (2013) provided evidence for the first time of key ecological roles based on the diversified activation for the OR19a receptor. Dweck et al. (2013) demonstrated that this subunit is necessary and sufficient to regulate the *D. melanogaster* ovipository preference for citrus, which represents an ancestral trait for *D. melanogaster*. Presumably, this results from an adaptation to fruits found within the native African habitat, pricing the *D. melanogaster*’s fitness, since some of the various terpenoids active on OR19a such as limonene and valencene repel the *Leptopilina boulardi* endoparasitoid. Based on the results of the work of Dweck et al. (2013), another study investigated limonene and valencene as oviposition stimulants, comparing *D. melanogaster* with *Drosophila simulans* and the specialist *Drosophila sechellia*, unveiling *D. sechellia* flies being indifferent to or avoiding oviposition on substrates containing either of these chemicals, with suppressed egg-laying at high stimulus concentrations (Alvarez-Ocana et al., 2023). These data seem to be coherent with evidence of a reduced number of OR19a neurons in *D. sechellia* antennae, when compared with *D. melanogaster* and *D. simulans* (Dr. Alvarez-Ocana, personal communication). Taken together with the findings of Dweck et al. (2013), the findings of Alvarez-Ocana et al. (2023) may suggest that in the genus *Drosophila*, the OR19a olfactory pathways play a host-recognition role for insects with a wide host range, rather than for stringent specialists, such as *D. sechellia*. This is in accordance with our findings of a wide tuning that we demonstrated for the OR19A1 subunit of *D. suzukii* (Figure 3B), given the high degree of polyphagia renowned for this insect (Asplen et al., 2015).

Not surprisingly, some of the most active terpenes that we identified binding OR19A1 (Figure 3B) are part of the essential oil content from the mandarin citrus plant *Citrus reticula.* Among these terpenoids, α-humulene, β-myrcene, and γ-terpinene are present in low percentages (0.06-0.11%), α-pinene is one among the most abundant (1.75%), and D-limonene is the most abundant (85.1%) (Boughendjioua et al., 2021). Among various Californian crops, *Citrus reticulata* represents an alternative ovipositional and/or reproductive host for *D. suzukii*, on which fruits high percentages of eggs develop to adults (Wang et al., 2019). While these pieces of evidence seem to indicate that the ligands that we found active on DsuzOR19A1 are most likely attractive for *D. suzukii*, further behavioral investigations are needed to explore the possible role of this receptor and its discrimination in the attraction between males and females. Essential oils from mandarin were also demonstrated to be repulsive on *D. suzukii*, both when tested in dual choice and in oviposition behavioral assays (Bedini et al., 2020).

To shed light on the behavioral importance for terpenoids on *D. suzukii*, future experiments may take advantage from the ongoing development of CRISPR-cas9 technologies for this insect (Ni et al., 2021). Comparison between wild-type flies and CRISPR-knock-out mutants for the OR19A1 locus may search for key ligands among the ones that we have found active to help the development of new ways to interfere with the behavior of *D. suzukii*. Future studies may also investigate the ecological relevance of these various compounds, attempting to unveil their relations with oviposition, food preference, or more complex interactions with sensing modalities from the natural enemies of this Drosophilid.

## Conclusion

Up to our knowledge, this is first time where the heterologous expression of GPCR-ORs has been attempted into transgenic neurons of *D. melanogaster*. Although we did not demonstrate binding of ligands, it seems that these subunits re-establish ab3A spiking in empty neurons from *Drosophila*. Since the aim of this study was mostly comparative, further investigations *in situ* are needed to address whether these subunits may be targeted to the dendrites of neurons and if such re-established ab3A spiking would somehow relate with possible metabotropic interactions with local cation channels.

Similarly, we attempted the expression of IR subunits into the ab3A neurons of *D: melanogaster*. Although additional studies are necessary, our evidence of a complete lack of spiking for both subunits from *C. pomonella* and the phylogenetically closer *D. suzukii* (Walker et al., 2023) and for the ci-expressed *D. melanogaster* IR84a (Abuin et al., 2011) may indicate that ab3As is a less reliable tool for attempting IR expression.

With this, we recognize that efforts from this study to heterologously express mammalian GPCR and insect IRs into ab3A neurons resulted in negative data. However, we are convinced that despite being negative, these findings are important to prevent other groups from attempting similar experiments in vain.

Contrary to these negative data, evidence from the functional expression of both DsuzOR19A subunits and the deorphanization from DsuzOR19A1 add to our previous efforts in decrypting sensing modalities of *D. suzukii*. While we demonstrated tuning for a subunit with a female-specific antennal bias to a wide range of ligands, with sesquiterpene enhancing a tonic firing pattern, future studies may explore a wider asset of ligands to attempt succeeding in DsuzOR19A2 deorphanization. The function of OR19A2 remains to be elucidated. Our evidence *in situ* suggests the importance of achieving DsuzOR19A2 deorphanization given the possible implication of ligands active on this subunit in male-specific olfactory sensing modalities.

## Data Availability

The original contributions presented in the study are included in the article/Supplementary Material; further inquiries can be directed to the corresponding author.
