# Supplementary figures and images for: Heterologous investigation of metabotropic and ionotropic odorant receptors in ab3A neurons of Drosophila melanogaster
Source: Front Mol Biosci. 2024 Jan 25;10:1275901. doi: 10.3389/fmolb.2023.1275901 (PMC10853936; doi:10.3389/fmolb.2023.1275901)

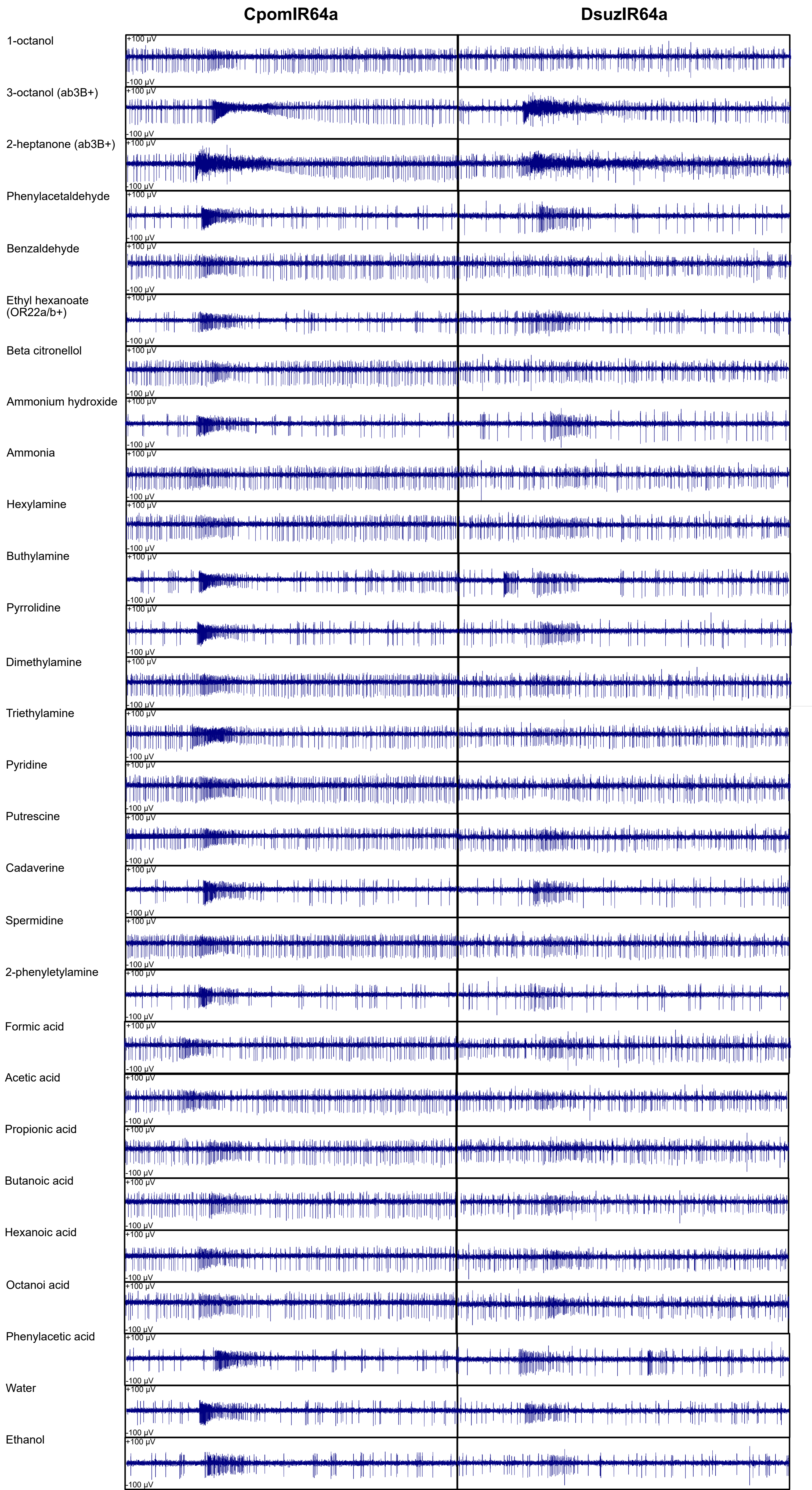

Supplement: Supplementary file 1 [file DataSheet2.PDF]

Basic firing, genotype *w*; +; +

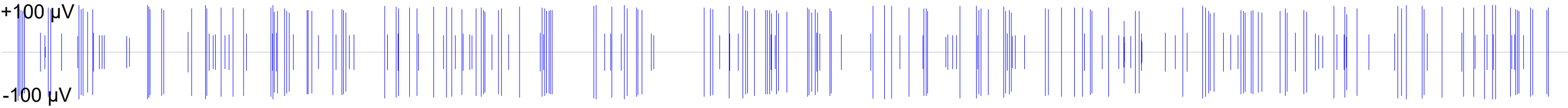

Basic firing, genotype *w*;  $\Delta$ *Halo*; *pUAS-DsuzOR19A2/p22a-Gal4*

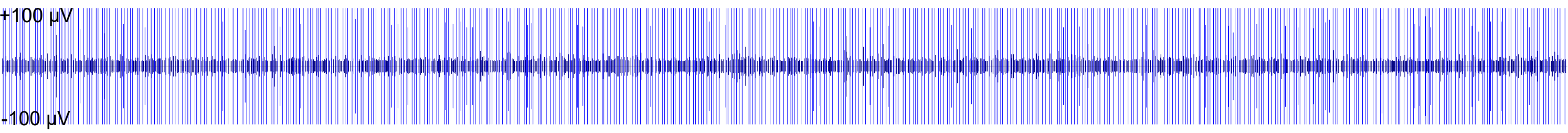

B

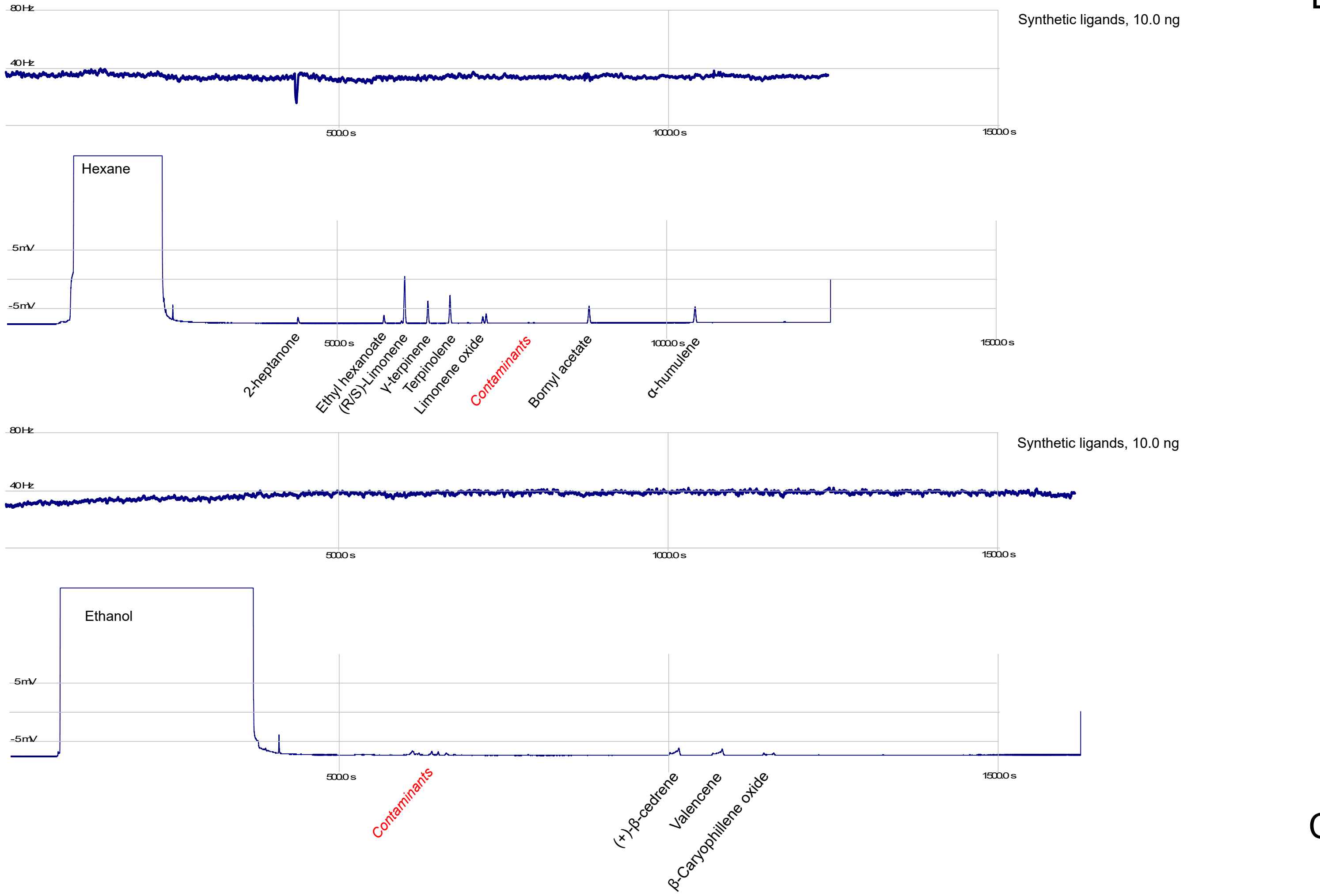

C

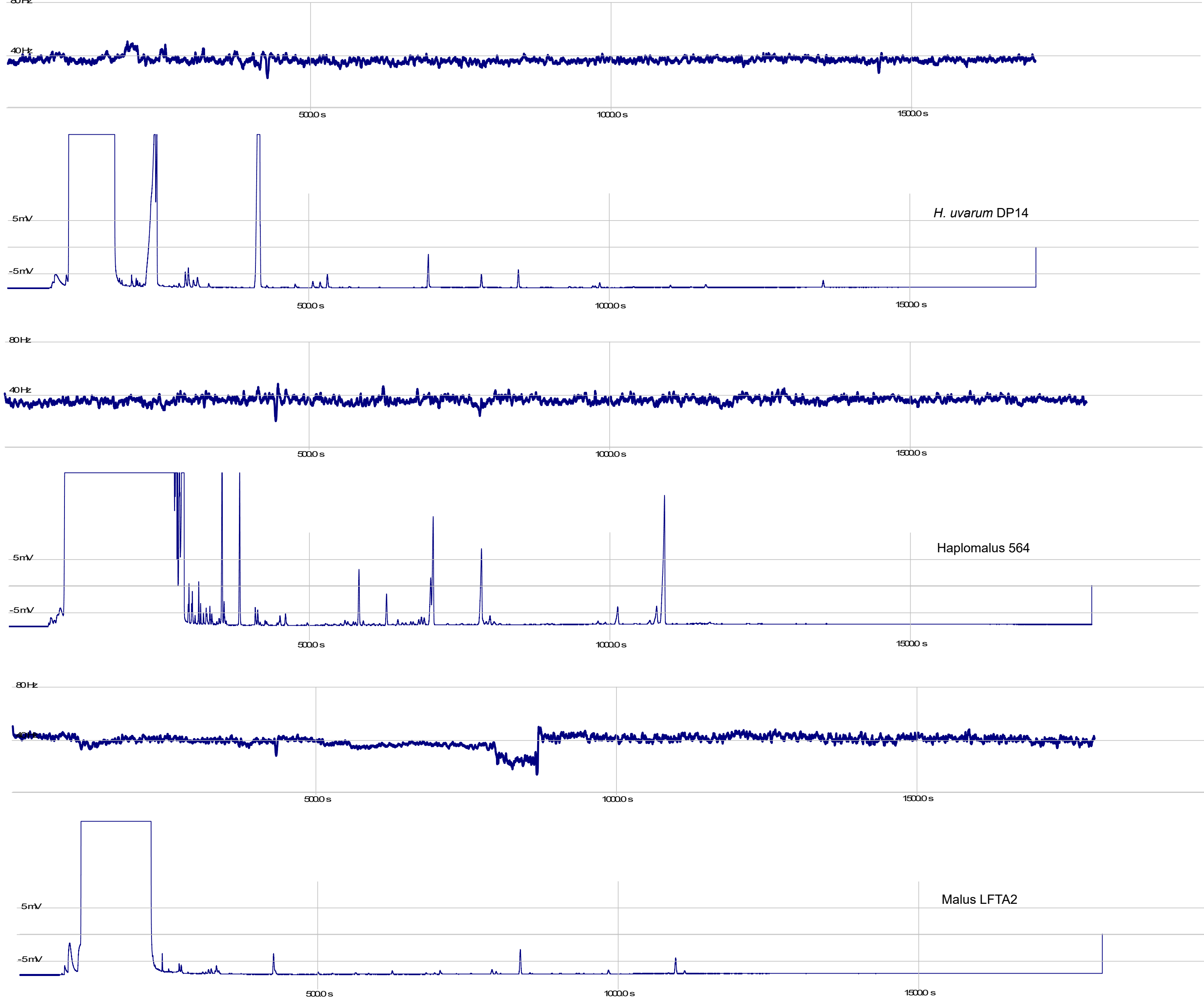

Supplement: Supplementary file 3 [file DataSheet4.PDF]

A

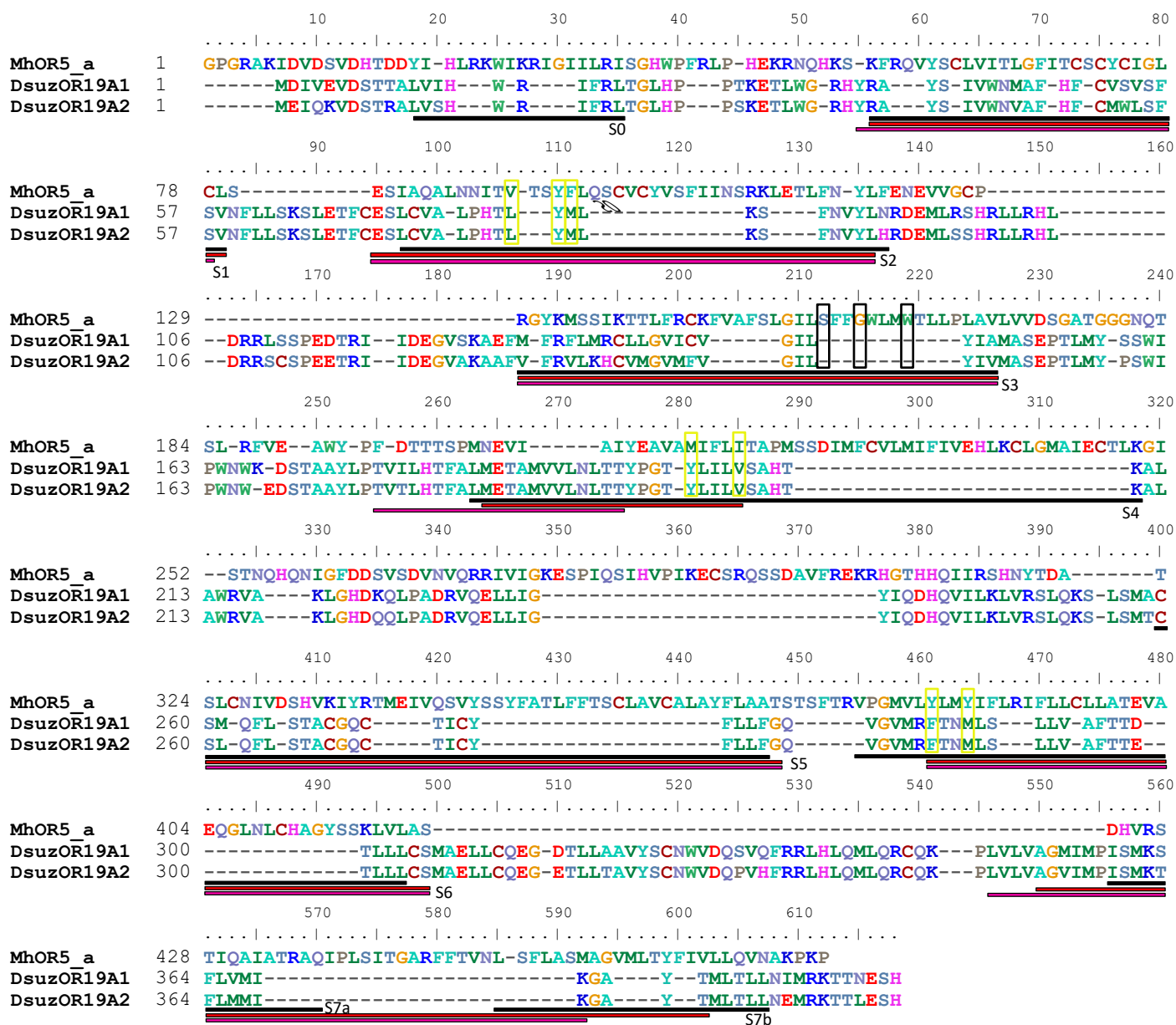

B

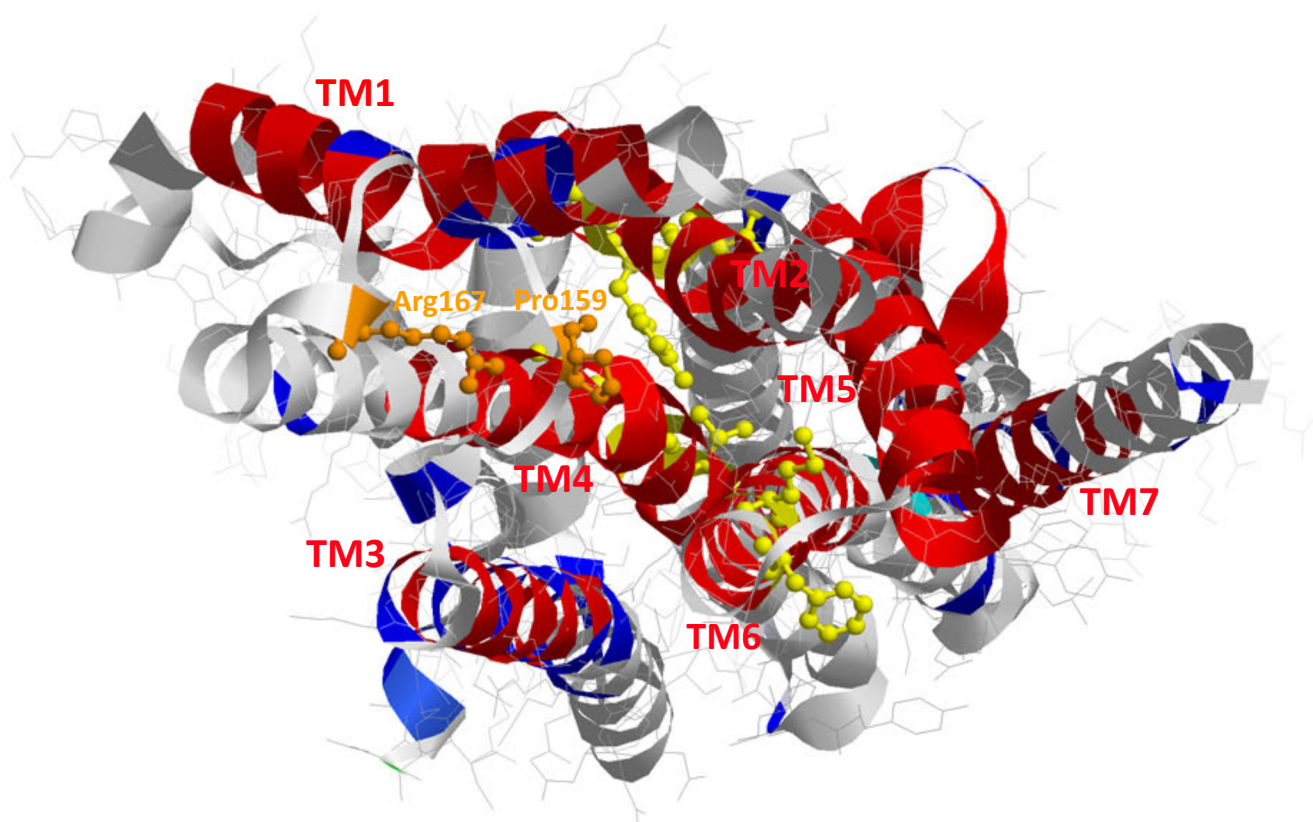

Supplement: Supplementary file 4 [file DataSheet6.PDF]
