# Supplementary material for: Heterologous investigation of metabotropic and ionotropic odorant receptors in ab3A neurons of Drosophila melanogaster
Source: Front Mol Biosci. 2024 Jan 25;10:1275901. doi: 10.3389/fmolb.2023.1275901 (PMC10853936; doi:10.3389/fmolb.2023.1275901)
Supplement: Supplementary file 7 [file DataSheet5.PDF]

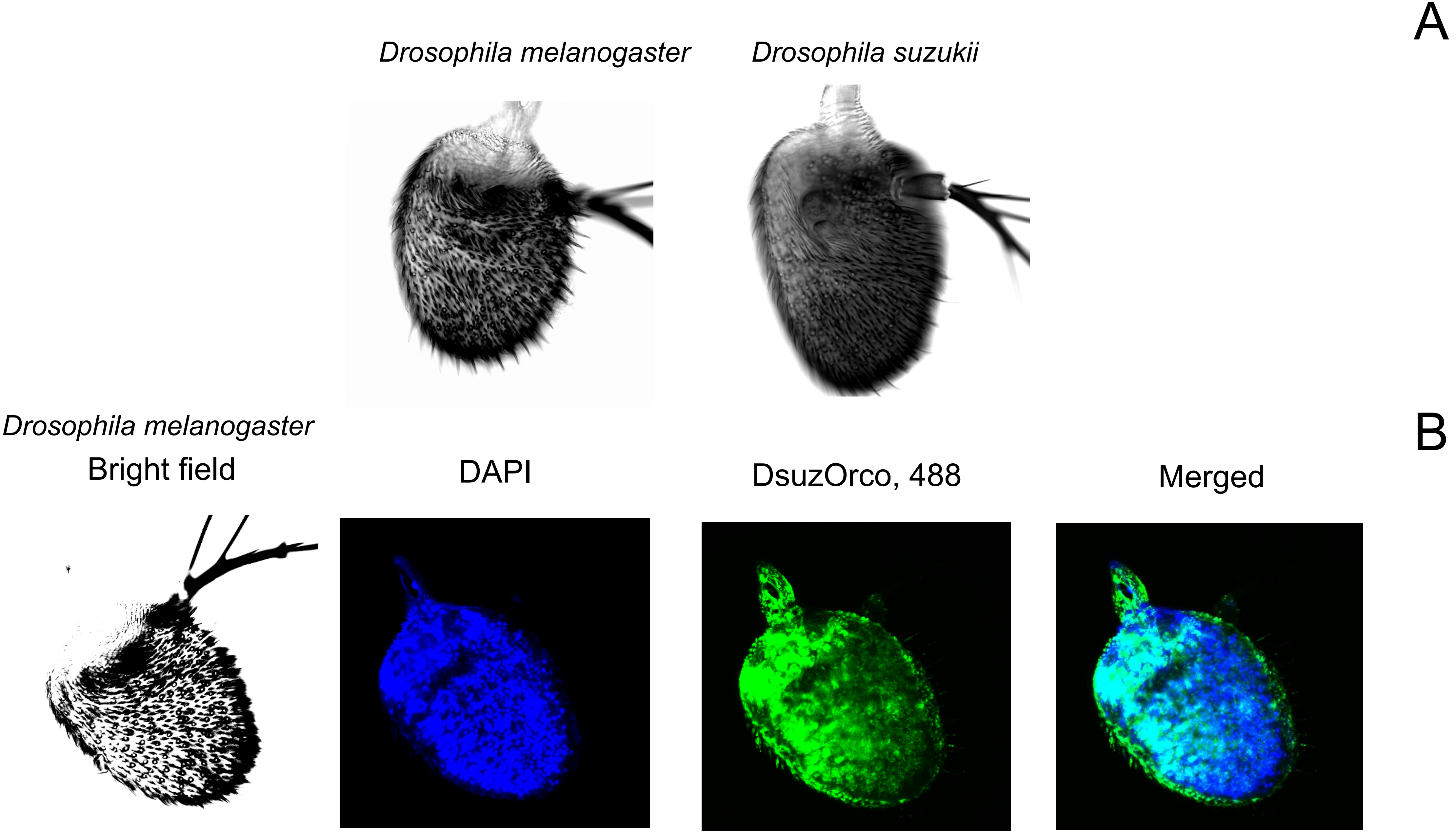
A
Drosophila melanogaster
Drosophila suzukii
B
Drosophila melanogaster
Bright field
DAPI
DsuzOrco, 488
Merged
