# Supplementary material for: Heterologous investigation of metabotropic and ionotropic odorant receptors in ab3A neurons of Drosophila melanogaster
Source: Front Mol Biosci. 2024 Jan 25;10:1275901. doi: 10.3389/fmolb.2023.1275901 (PMC10853936; doi:10.3389/fmolb.2023.1275901)
Supplement: Supplementary file 9 [file DataSheet1.docx]

**Supplementary captions**

**Supplementary Figure S1** **[Image 1]** (**A**) Schematic representation of crossings to generate transgenic lines for SSR. Note: for the various transgenic lines, the transgene is indicated in **bold**; the *pUAS-IR8a,pOR22a-Gal4^KI^* genotype obtain through the single fly PCR screening from B is indicated in red. (**B**) Gel electrophoresis from single fly PCR performed using different primer pairs indicated in Supplementary Table S1. Single fly PCR was conducted to test the co-existence of *pUAS-IR8a* and *pOR22a-Gal4^KI^* constructs upon their recombination from the parental lines *w;pUAS-IR8a^(w+)^;+* and *w;pOR22a-Gal4;+*. 1-3: DNA from *w;pOR22a-Gal4^KI^,pUAS-IR8a/CyO;TM2/TM6B* recombined genotypes; 4-5: Parental lines (*w;pUAS-IR8a^(w+)^;+* and *w;pOR22a-Gal4^KI^;+* respectively) 6: negative control genotype *w;+;+* ; 7: yeast sample collected from insect vial (negative control). Ladder: O’GeneRuler Express (Thermo Fisher Scientific).

**Supplementary Figure S2 [Data Sheet 2]** Ligand screening on transgenic *D. melanogaster* expressing IR64a subunits in ab3A neurons. Ligand screening reporting absence of ab3A-spiking for the IR-specific ligands from Table 1. Note: for most of the stimuli, the activation of ab3B resembles the effect of the solvents (water/ethanol) suggesting such effect being associated with an artifact. Note: rare ab3A spikes were visible only in some cases providing strong ab3B-activators, including 2-heptanone and 3-octanol, as reported in Figure 1. Left: *w;pUAS-IR8a,pOR22a-Gal4;pUAS-****CpomIR64a*** - [N = 4]. Right: *w;pUAS-IR8a,pOR22a-Gal4;pUAS-****DsuzIR64a*** - [N = 3].

**Supplementary Figure S3** **[Data Sheet 3]** (**A**) Example of phasic and tonic effects when providing 25 μg of S-limonene and valencene on *D. melanogaster* ab3A neurons expressing DsuzOR19A1. Note: compounds were provided in the course of the same recording. (**B**) Frequency plots (Hz) and spike trains (μV) distinguishing ab3A (blue) from ab3B effects (black) when each ligand was tested on *D. melanogaster* expressing DsuzOR19A1 (replicate 4) and DsuzOR19A2 (replicate 1) (SSR = 80 Hz, Bin-width = 25 ms, Smooth filter = 2 Taps). Compounds enhancing tonic effects for DsuzOR19A1 are indicated in red. Ligands are listed according with Supplementary Data File 1.

**Supplementary Figure S4 [Data Sheet 4]** (**A**) Spike-train comparison of ab3A neurons expression of *w;+;+* mutants (above) and DsuzOR19A2 transgenic (below) based on the *∆halo* system. Note: as in Figure 3, in all the experiments we observed higher firing rate for OR19A2. (**B**) Partial GC-SSR screening comparing 10 ng doses of various ligands: some ligands were diluted in hexane, others in ethanol as indicated in the figure. Note: decrement of spiking in correspondence with 2-heptanone is related with ab3B activation. Evidence of a similar effect when testing headspace collections suggested rather the presence of 2-heptanone or of an ab3B activator within the headspace. However, this compound is inactive on ab3A and it is not of interest. (**C**) GC-SSR experiments testing headspace collected from *Hanseniaspora uvarum* (*H. uvarum* DP14, injection: 1.5 μL (Cattaneo et al., 2022) and headspace collected from apple (Haplomalus 564, injection: 5.0 μL; LFTA, injection: 3.0 μL) that will be part of a different project (Cattaneo et al. in preparation). Note: GC-SSR experiments were conducted following protocols described in Cattaneo et al. (2022); contaminants are indicated in red.

**Supplementary Figure S5** **[Data Sheet 5]** Comparison of the antennae of *D. melanogaster* and *D. suzukii*. (A) Bright field showing an antenna sample from single insects; note the slight differences in shapes between the two species. (B) Staining with DsuzOrco probe (Cattaneo et al., 2022), comparing the bright field with dapi and 488 of a *D. melanogaster* antennae.

**Supplementary Figure S6** **[Data Sheet 6]** Identification of further amino acids with possible involvement in the ligand binding pocket of DsuzOR19As. (A) Polypeptide sequence alignment among OR5a of *Machilis hrabei* (Archaeognatha: Machilidae) PDB: 7LIC_A, *D. suzukii* OR19A1 and *D. suzukii* OR19A2. Note: despite del Mármol et al. (2021) showed a full-length *M. hrabei* OR5a polypeptide sequence, PDB: 7LIC_A is provided with 5 N-terminal amino acids (GPGRA) replacing Met1, indicating that the subunit that they have deposited refers most likely to an incomplete protein. Transmembrane domains are indicated with black underlines for *M. hrabei* (S0-S7a,b), and with red and magenta underlines for DsuzOR19A1 and DsuzOR19A2 (S1-S7, Figure 4). Amino acids involved in forming the binding pocket as in Marmol et al. (2021) are highlighted with yellow squares. Residues that our polypeptide sequence alignment did not identified are highlighted with black squares. (B) 3D structure of DsuzOR19A1 highlighting in yellow the transmembrane residues from A, which we identified between TM2 and TM6. Colors are used as in Figure 4B. Residues laying within the TM1-TM6 pocket, like Pro159 (Ser159 for DsuzOR19A1) and Arg167 (Lys167 for DsuzOR19A1), are indicated as in Figure 4C. Note the proximity of Pro159 with some of the residues highlighted in yellow.

**Supplementary Data File S1 [Table 1]** Spike counting (spikes/sec), statistical analysis with box-plots from Figures 2 B and 3 B. For DsuzOR19A1 we indicated also the effect, rather phasic (P) or Tonic (T). For DsuzOR19A2 once we reached three replicates we observed that the highest responses averaged values proximal to 8 spikes/seconds (3-octanol), which were even lower than the limit of significance observed for DsuzOR19A1 (i.e. citral = 8.83 ± 4.16 spikes/sec). From these evidences, we decided to interrupt recordings concluding that the SSR-investigation on DsuzOR19A2 deserved to address rather choices for different ligands or heterologous expression towards different OSNs.

**Supplementary Data File S2 [Table 2]** Neuronal counting on *D. suzukii* male and female antennae stained with OR19A1- and OR19A2-probes from Figure 3 C. The file shows neuronal counting and statistical parameters from a heteroscedastic one-tailed two-samples T-Test (OR19A1: *p* = 0.03394) and from a Mann-Whitney U-test (OR19A2: *p* = 0.004), both tests: ɑ = 0.05. Parameters from the box plot analysis of the number of counted neurons are also reported.

**Supplementary Table S1 [Table 3]** Primers used to test presence of constructs in single-fly PCR shown in Supplementary Figure S1 B.
