# Supplementary material for: Heterologous investigation of metabotropic and ionotropic odorant receptors in ab3A neurons of Drosophila melanogaster
Source: Front Mol Biosci. 2024 Jan 25;10:1275901. doi: 10.3389/fmolb.2023.1275901 (PMC10853936; doi:10.3389/fmolb.2023.1275901)
Supplement: Supplementary file 10 [file Image1.pdf]

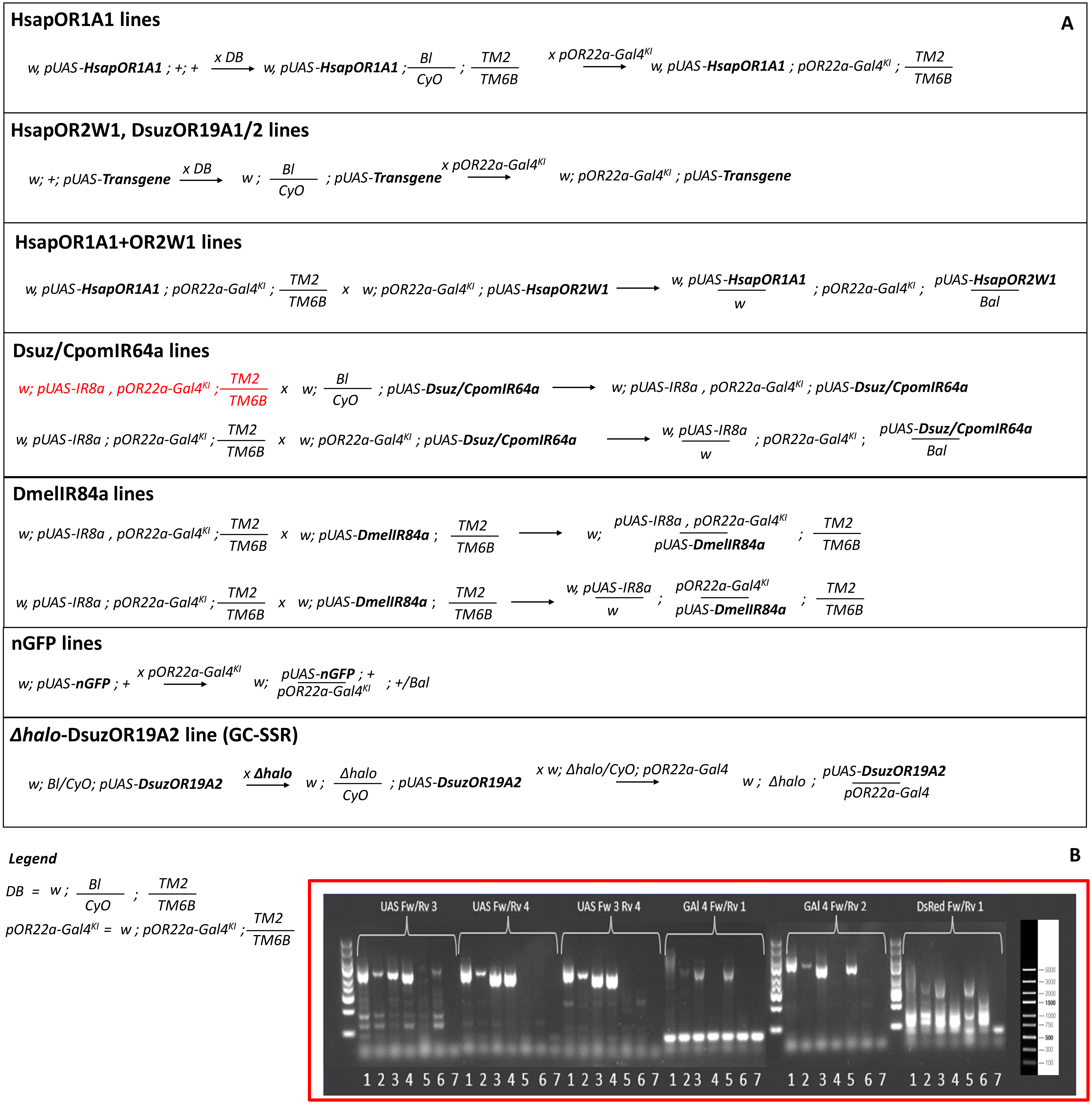

A
HsapOR1A1 lines
w, pUAS-HsapOR1A1 ; +; +
x DB
w, pUAS-HsapOR1A1 ; Bl / CyO ; TM2 / TM6B
x pOR22a-Gal4KI
w, pUAS-HsapOR1A1 ; pOR22a-Gal4KI ; TM2 / TM6B
HsapOR2W1, DsuzOR19A1/2 lines
w; +; pUAS-Transgene
x DB
w ; Bl / CyO ; pUAS-Transgene
x pOR22a-Gal4KI
w; pOR22a-Gal4KI ; pUAS-Transgene
HsapOR1A1+OR2W1 lines
w, pUAS-HsapOR1A1 ; pOR22a-Gal4KI ; TM2 / TM6B
x
w; pOR22a-Gal4KI ; pUAS-HsapOR2W1
w, pUAS-HsapOR1A1 / w ; pOR22a-Gal4KI ; pUAS-HsapOR2W1 / Bal
Dsuz/CpomIR64a lines
w; pUAS-IR8a , pOR22a-Gal4KI ; TM2 / TM6B
x
w; Bl / CyO ; pUAS-Dsuz/CpomIR64a
w; pUAS-IR8a , pOR22a-Gal4KI ; pUAS-Dsuz/CpomIR64a
w, pUAS-IR8a ; pOR22a-Gal4KI ; TM2 / TM6B
x
w; pOR22a-Gal4KI ; pUAS-Dsuz/CpomIR64a
w, pUAS-IR8a / w ; pOR22a-Gal4KI ; pUAS-Dsuz/CpomIR64a / Bal
DmelIR84a lines
w; pUAS-IR8a , pOR22a-Gal4KI ; TM2 / TM6B
x
w; pUAS-DmelIR84a ; TM2 / TM6B
w; pUAS-IR8a , pOR22a-Gal4KI / pUAS-DmelIR84a ; TM2 / TM6B
w, pUAS-IR8a ; pOR22a-Gal4KI ; TM2 / TM6B
x
w; pUAS-DmelIR84a ; TM2 / TM6B
w, pUAS-IR8a / w ; pOR22a-Gal4KI / pUAS-DmelIR84a ; TM2 / TM6B
nGFP lines
w; pUAS-nGFP ; +
x pOR22a-Gal4KI
w; pUAS-nGFP ; + / pOR22a-Gal4KI ; +/Bal
Δhalo-DsuzOR19A2 line (GC-SSR)
w; Bl/CyO; pUAS-DsuzOR19A2
x Δhalo
w ; Δhalo / CyO ; pUAS-DsuzOR19A2
x w; Δhalo/CyO; pOR22a-Gal4
w ; Δhalo ; pUAS-DsuzOR19A2 / pOR22a-Gal4
Legend
DB = w ; Bl / CyO ; TM2 / TM6B
pOR22a-Gal4KI = w ; pOR22a-Gal4KI ; TM2 / TM6B
B
UAS Fw/Rv 3
UAS Fw/Rv 4
UAS Fw 3 Rv 4
GAl 4 Fw/Rv 1
GAl 4 Fw/Rv 2
DsRed Fw/Rv 1
1 2 3 4 5 6 7 1 2 3 4 5 6 7 1 2 3 4 5 6 7 1 2 3 4 5 6 7 1 2 3 4 5 6 7 1 2 3 4 5 6 7
5000
3000
2000
1500
1000
750
500
300
100
