# Supplementary material for: Heterologous investigation of metabotropic and ionotropic odorant receptors in ab3A neurons of Drosophila melanogaster
Source: Front Mol Biosci. 2024 Jan 25;10:1275901. doi: 10.3389/fmolb.2023.1275901 (PMC10853936; doi:10.3389/fmolb.2023.1275901)
Supplement: Supplementary file 6 [file DataSheet3.PDF]

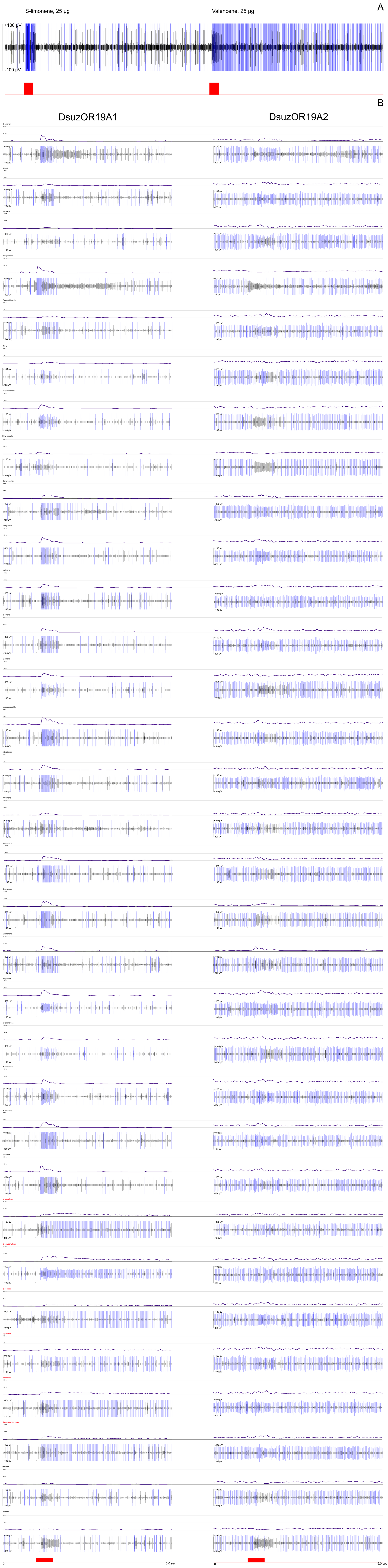
A
S-limonene, 25 µg
Valencene, 25 µg
+100 µV
-100 µV
B
DsuzOR19A1
DsuzOR19A2
0
5.0 sec
